# Empagliflozin–pirfenidone dual therapy improves cardiac function and structure in a preclinical two-hit HFpEF model

**DOI:** 10.3389/fphar.2026.1781866

**Published:** 2026-06-03

**Authors:** Yuexin Yu, Yaping Xu, Jinfu Chen, Yao Yao, Yingtian Liu, Tan Huang, Shaolong Yang, King-Hwa Ling, Jacques P. Guyette, Bakiah Shaharuddin, Zhikun Guo, Jun Jie Tan

**Affiliations:** 1 Zhengzhou Seventh People’s Hospital, Henan, China; 2 Advanced Medical and Dental Institute, Universiti Sains Malaysia, Penang, Malaysia; 3 Institute of Biological Therapy, Henan Academy of Innovations in Medical Science, Henan, China; 4 Henan Key Laboratory of Medical Tissue Regeneration, Xinxiang Medical University, Xinxiang, China; 5 The First Affiliated Hospital of Zhengzhou University, Henan, China; 6 Neurobiology and Genetics Group, Medical Genetic Unit, Department of Biomedical Sciences, Faculty of Medicine and Health Sciences, Universiti Putra Malaysia, Selangor, Malaysia; 7 School of Nursing, Zhengzhou Railway Vocational and Technical College, Henan, China; 8 Harvard Medical School, Boston, MA, United States; 9 Zhejiang Tianyuan Biotechnology Co. Ltd., Ningbo, Zhejiang, China

**Keywords:** cardiac fibrosis, dual therapy, empagliflozin, heart failure with preserved ejection fraction, pirfenidone, sodium–glucose co-transporter-2 inhibitor, compartmentalized synergy

## Abstract

**Background:**

Heart failure with preserved ejection fraction (HFpEF) accounts for more than half of heart failure cases and is associated with substantial morbidity and mortality. Although sodium–glucose cotransporter-2 inhibitors (SGLT2is) have shown efficacy in reducing HFpEF hospitalizations, the disease’s multi-pathway pathogenesis often limits the effectiveness of single-target interventions. This study examines whether the cardiovascular benefits of empagliflozin (EMPA) can be synergistically enhanced by co-administration with the targeted anti-fibrotic agent pirfenidone (PFD).

**Aims:**

We hypothesized that simultaneous modulation of metabolic stress and pro-fibrotic signaling would lead to synergistic structural and functional recovery in a severe HFpEF phenotype.

**Methods:**

HFpEF was induced in Sprague–Dawley rats (n = 16/group) using a two-hit model [NG-nitro-L-arginine methyl ester (L-NAME) and a high-fat diet (HFD)] over 5 weeks. The rats subsequently received daily oral EMPA (0.35 g/kg/d), PFD (0.3 g/kg/d), or combination therapy (EMPA + PFD) for 4 weeks. Treatment efficacy was evaluated via echocardiography, histopathology, and exercise tolerance testing. Further insights into the underlying transcriptional mechanisms were gained through RNA sequencing and semi-quantitative Western blotting. Bliss independence analysis was used to analyze pharmacological synergy.

**Results:**

Co-treatment with EMPA + PFD significantly enhanced diastolic indices, left ventricular ejection fraction (LVEF), fractional shortening (FS), and cardiac output (CO) compared with controls and monotherapy groups (p < 0.05). Histological analysis revealed reduced cardiomyocyte hypertrophy and lower collagen deposition, particularly in the endocardium. Although additive improvement in systemic hemodynamics was observed, EMPA + PFD co-treatment exerted compartmentalized synergy within the myocardium. The combination reversed cellular hypertrophy and deep fibrosis, restoring diastolic and systolic function (p < 0.05). Transcriptomic profiling revealed that local myocardial rescue was mediated by gene networks linked to protein kinase C-activating G-protein-coupled receptor signaling, which blocked the downstream PKC/NF-κB inflammatory pathway and transforming growth factor-beta (TGF-β)-driven fibrosis, thereby explaining the molecular basis of functional improvement.

**Conclusion:**

Dual therapy with EMPA and PFD achieves compartmentalized synergy, effectively eliminating cellular hypertrophy and deep fibrosis, thereby restoring cardiac mechanics. These findings provide mechanistic proof of concept that multi-axis metabolic and antifibrotic combinations can disrupt the complex pathological cycle of HFpEF.

## Highlights

- Co-administration of empagliflozin and pirfenidone synergistically targets metabolic and profibrotic axes in a two-hit HFpEF model.
- The combination exerts compartmentalized synergy, reversing cellular hypertrophy and fibrosis to restore cardiac mechanics.
- The synergistic benefits are driven by the simultaneous targeting of the PKC/NF-κB profibrotic cascade and metabolic/hemodynamic stress by EMPA + PFD.

## Introduction

Heart failure with preserved ejection fraction (HFpEF) is an emerging clinical health threat affecting more than half of all heart failure (HF) patients in the United States (Becher et al., 2022). The 2-year rate of hospitalization for HF or all-cause death is reported to be 35% in HFpEF, with a corresponding 2-year mortality rate of 14% (Lam et al., 2018). This imposes a significant socioeconomic burden on healthcare systems. Although patients with HFpEF have “preserved” systolic function (with an ejection fraction greater than 50%), the disease notably shares similar, if not worse, mortality and cumulative survival rates with heart failure with reduced ejection fraction (HFrEF) (Shahim et al., 2023). This limited survival benefit is reflected in most clinical trials evaluating therapeutic agents indicated for HFrEF, including statins, renin–angiotensin–aldosterone system inhibitors, angiotensin II receptor blockers, and mineralocorticoid antagonists (van de Bovenkamp et al., 2025). The persistent treatment gap underscores the urgent need to develop effective therapies specifically tailored for HFpEF.

Progressive cardiac fibrosis is predominant in HFpEF and is a key factor in the deterioration of diastolic reserve and worsening cardiac performance (Su et al., 2014). One study found that cardiac fibrosis was present in 93% of HFpEF patients, with a median left ventricular ejection fraction (LVEF) of 65% (Hahn et al., 2020). Accumulating data demonstrated a direct correlation between myocardial fibrosis burden and poor prognosis in HFpEF patients (Zhang et al., 2024). Given the increased risk of arrhythmias, hospitalization, and mortality, cardiac fibrosis can serve as a key marker for risk stratification and mortality prediction in HFpEF management.

Sodium–glucose co-transporter-2 inhibitors (SGLT2is) are established treatments for type 2 diabetes and chronic kidney disease, recognized for their efficacy in improving vascular resistance and fluid overload (Mayne et al., 2024). Beyond their metabolic benefits, recent clinical trials have demonstrated that SGLT2i can significantly reduce the risk of cardiovascular death or HF hospitalization in patients with an LVEF >40%, regardless of the diabetes status (Anker et al., 2021; Solomon et al., 2022; Wiviott et al., 2019). These findings have led to a Class 2a recommendation for SGLT2i in the treatment of HFpEF within the latest AHA/ACC guidelines (Heidenreich et al., 2022a). Although preclinical studies suggested that SGLT2is exert anti-fibrotic properties (Santos-Gallego et al., 2021; Mason et al., 2021; Yang et al., 2022; Lee et al., 2022), this has appeared modest in clinical settings, and its contribution to patient outcomes remains uncertain (Nicholls and Nerlekar, 2021). This observation is consistent with the lack of significant reductions in circulating cardiac fibrosis biomarker levels following SGLT2i therapy (Lebedev et al., 2021) despite the established correlation between these markers and both the extent of cardiac fibrosis and patient prognosis (Ibrahim et al., 2016). Hence, the antifibrotic effects of SGLT2i are likely non-canonical as SGLT2 receptors are minimally expressed in human cardiac tissue compared to SGLT1, indicating limited direct action on cardiomyocytes or fibroblasts (Mourad et al., 2025). Other proposed off-target effects, such as inhibition of sodium–hydrogen exchanger 1 (NHE1) in cardiomyocytes, remain unconfirmed in human HFpEF trials (Chen et al., 2024).

Pirfenidone (PFD) is a synthetic pyridine drug known for its inhibitory action on transforming growth factor β1, which underlies its anti-fibrotic properties. It is FDA-approved for treating idiopathic pulmonary fibrosis in humans, while accumulating preclinical evidence suggests its therapeutic potential in cardiac inflammation, hypertrophy, and fibrosis (Yamagami et al., 2015). These findings prompted the initiation of the PIROUETTE trial, a randomized, double-blinded, placebo-controlled Phase II trial assessing the efficacy and safety of PFD in patients with HFpEF over 52 weeks (Lewis et al., 2021). The trial demonstrated a significant reduction in myocardial extracellular volume and improved N-terminal pro-B-type natriuretic peptide (NT-proBNP) levels following PFD treatment, as well as significant improvements in LVEF and global longitudinal strain.

We demonstrated that co-treatment with empagliflozin (EMPA) and the antifibrotic agent pirfenidone provides an additive benefit over cardiac function and reversal of cardiac fibrosis in a two-hit HFpEF rat model.

## Methods

### Animal study

All animal experiments and protocols were conducted in accordance with the NIH Guide for the Care and Use of Laboratory Animals and reviewed and approved by the Institutional Animal Care and Use Committee of Zhengzhou Seventh People’s Hospital, Henan, China (approval reference No: 2021–1203017). Twenty-four-week-old Sprague–Dawley rats (300–350 g) were housed in the Henan Key Laboratory of Cardiac Remodeling and Transplantation at the hospital for 1 week before the commencement of experiments. The animals were maintained under controlled conditions with a temperature range of 20 °C–25 °C, a humidity range of 50%–65%, and a 12-h light/dark cycle, with unrestricted access to food and water. HFpEF was induced in the rats using NG-nitro-L-arginine methyl ester (L-NAME, GA11233, GlpBio, 0.5 g/L, dissolved in drinking water, with pH adjusted to 7.4) and a high-fat diet (HFD, 60% kilocalories from lard, D12492, Teklad) for a duration of 5 weeks. Control rats were not administered L-NAME in their drinking water. HFpEF characteristics in the rats were confirmed after 5 weeks.

Subsequently, HFpEF rats were randomly assigned to receive daily oral doses of a supratherapeutic dose of EMPA (0.35 g/kg/day; Aladdin Scientific, Shanghai, China), PFD [0.3 g/kg/day (Yamazaki et al., 2012); Aladdin Scientific, Shanghai, China], or a combination of both. EMPA was suspended in phosphate-buffered saline (PBS) at 35 mg/mL, vortexed vigorously, and sonicated for 10 min immediately before oral gavage (10 mL/kg) to ensure uniform dispersion; dosing was completed within 15 min of preparation. HFpEF rats receiving PBS alone served as the vehicle control group. Each group comprised 16 rats with an equal sex distribution. Animals were euthanized at weeks 5 and 9 via carbon dioxide inhalation before organ harvesting for histochemical analysis or RNA sequencing.

### Kaplan–Meier survival curve analysis, log-rank test, and hazard ratios

Treated rats were observed, and survival was recorded over 4 weeks following HFpEF induction. Kaplan–Meier survival, Mantel–Cox log-rank test, and hazard ratios were calculated using Python 3.13 with lifelines and CoxPHFitter libraries, based on pooled male and female survival data from 64 rats (8 male and 8 female rats per group, across four treatment groups: Control, EMPA, PFD, and EMPA + PFD). Time-to-event data (days to death or censoring) and event status (1 = death; 0 = censored) were entered into a Cox proportional hazards regression model and analyzed using GraphPad Prism (version 10) to assess the effects of treatment, gender, and their interaction on survival. The model included Treatment (reference: Control), Gender (reference: Male), and Treatment × Gender interaction terms, with exact estimation to handle associations. Parameter estimates (β) and hazard ratios (HR = e^β) with 95% confidence intervals were calculated to quantify the impact of each predictor on the hazard rate. Model fit was evaluated using the log-likelihood ratio test, and the proportional hazards assumption was assessed via diagnostic plots. Due to high censoring (73.44%, 47/64 rats), individual Kaplan–Meier survival curves were generated to visualize differences in survival across groups. All analyses accounted for the study’s design, with significance set at p < 0.05.

### Echocardiography

Transthoracic echocardiography was performed at baseline (0 W), 5^th^ week (5 W), and 9^th^ week (9 W) under general anesthesia with 1%–2% isoflurane, using a high-frequency ultrasound system (Vevo 2100; MS-250 transducer; VisualSonics, Toronto, Ontario, Canada). The cardiac systolic function was measured from M-mode scans at the parasternal long-axis section and the parasternal short-axis section. Heart rate (HR), left ventricular ejection fraction (LVEF), fractional shortening (FS), stroke volume (SV), cardiac output (CO), and cardiac mass were also measured. The measurements of diastolic function were obtained from apical four-chamber views using pulsed-wave imaging at the level of the mitral valve, including peak Doppler blood inflow velocity across the mitral valve during early diastole (E), peak Doppler blood inflow velocity across the mitral valve during late diastole (A), isovolumic relaxation time (IVRT), and E-wave deceleration time (DT). Analyses were performed offline using Vevo Lab software (version 5.5) by a single, blinded investigator. All representative echocardiographic images were obtained from male rats as statistical analysis revealed no significant sex differences.

### Blood pressure measurement

Blood pressure was measured using the tail-cuff method using a blood pressure meter (Softron, BP-2010A Series). All rats were trained to short-term restraint using a small-animal restrainer at 37 °C. Data were acquired under steady-state conditions to ensure their comfort. Blood pressure values were derived from six repeated measurements, and the mean was calculated to represent the blood pressure at the time of measurement.

### Exercise test

A treadmill exercise exhaustion test was performed. Rats from all groups were trained to run uphill at 20° on the treadmill, starting with a warm-up speed of 5 m/min for 4 min, after which the speed was increased to 14 m/min for 2 min. The speed was increased by 2 m/min every subsequent 2 min until the rat was exhausted, as indicated by the rat’s inability to resume running within 10 s of direct contact with an electric-stimulus grid. The running time was recorded, and the running distance was calculated.

### Fasting and postprandial blood glucose levels

The fasting blood glucose level (mg/dL) was tested after an overnight fast of 10 h. The postprandial blood glucose level was measured 120 min after intraperitoneal injection of 50% glucose solution (2 g/kg). Both levels were measured using a glucometer (ONETOUCH Ultra Vue, Johnson and Johnson, Shanghai, China).

### Blood biochemical analysis

Complete blood counts from blood samples collected from the inferior vena cava were analyzed using a blood analyzer (BC-7500CRP, Mindray North America).

### Histology and analysis

The extent of cardiac myocyte hypertrophy in the left ventricle was evaluated using wheat germ agglutinin (WGA) staining to measure cardiomyocyte cross-sectional area (CSA). Frozen left ventricular tissues were sectioned at 6 μm using a cryotome, fixed with 4% paraformaldehyde for 15 min at 37 °C, washed three times with HBSS (without phenol red), and then incubated with FITC-conjugated WGA (5–20 μg/mL in HBSS, Maokangbio, MP6325) for 10 min at room temperature. Afterward, the sections were washed three times with HBSS to remove the unbound dye and counterstained with DAPI (1 μg/mL, Sigma-Aldrich, D9542) for 5 min to visualize the nuclei. Fluorescence images were captured using a confocal microscope (Olympus FV3000) at ×20 magnification. For each heart, 10–15 randomly selected fields were analyzed. The cardiomyocyte CSA was quantified using ImageJ software by tracing the WGA-stained membrane boundaries of myocytes with clearly visible nuclei. Approximately 100–150 myocytes per animal were measured, and the average CSA was calculated for each group. All analyses were performed by a blinded investigator.

Heart sections were also stained using the Modified Masson’s Trichrome Stain Kit (G1346, Beijing Solarbio Science and Technology). Cardiac fibrosis was analyzed by determining the collagen volume fraction (CVF) from 15 fields at ×20 magnification, and images were quantified using ImageJ software. The lung wet-to-dry weight ratio (LWDR) was calculated to assess pulmonary congestion. The ratio of left ventricular weight to tibial length was measured as the left ventricular weight index (LVWI), serving as a morphometric index of cardiac hypertrophy severity in rats. All representative histological images were obtained from male rats as statistical analysis revealed no significant sex differences.

### RNA sequencing (RNA-Seq)

The left ventricles of the male rats were harvested, and total RNA was isolated using TRIzol reagent. Samples were sent to Beijing Genome Institute (BGI, China) for processing and analysis. RNA quality was assessed using an Agilent 2100 bioanalyzer, and RNA quantification was performed using the NanoDrop 2000 Spectrophotometer System. The sequencing data were preprocessed on the public server of the Galaxy web platform (usegalaxy.org) (Afgan et al., 2018). Fastq files were read and trimmed using Trim Galore (Krueger, 2021) for adapter sequences and reads below the average Phred score of 20. FASTQC was used to quality-test individual samples (Andrews, 2010). Sequences from passed samples were mapped to the rn6 rat reference genome using the HISAT2 tool and generated the corresponding BAM files (Kim et al., 2015). The htseq-count software was used to count aligned reads in BAM files that overlap features in GFF files (Anders et al., 2015). We used iDEP.96, an integrated web application for differential gene expression and functional ontology analyses (Ge et al., 2018). A total of 2,000 most variable genes were included for hierarchical clustering based on the correlation (average linkage) method. A cut-off Z score of 4 was used. DESeq2 (idep ver 0.96) was used to determine the differential gene expression between two groups, with an FDR cut-off of 0.05 and a minimum fold-change of 2 (Love et al., 2014). Enrichment analysis of differentially expressed genes (DEGs) based on GO terms for biological processes was also performed. RNASeq data were uploaded to the Gene Expression Omnibus (GEO) database (GSE277623).

### Western blotting for PKC and NF-κB activation

Left ventricular tissue samples were homogenized in ice-cold RIPA lysis buffer (Beyotime, P0013B) supplemented with protease inhibitor cocktail (Roche, 04693159001) and phosphatase inhibitor cocktail (Roche, 04906845001). Homogenates were centrifuged at 12,000 × g for 15 min at 4 °C, and the supernatant was collected. Protein concentrations were determined using the BCA protein assay kit (Thermo Scientific, 23225). Equal amounts of proteins (30 μg per lane) were separated using 10% SDS-PAGE and transferred onto polyvinylidene difluoride (PVDF) membranes (Millipore, IPVH00010). Membranes were blocked with 5% non-fat milk in Tris-buffered saline containing 0.1% Tween-20 (TBST) for 1 h at room temperature, followed by overnight incubation at 4 °C with primary antibodies: rabbit anti-Phospho-PKC (pan) (βII Ser660) (1:5000, Selleck, G6B3), rabbit anti-Phospho-NF-κB p65 (Ser536) (1:5000, Selleck, M4C8), and rabbit anti-GAPDH (1:1000, Selleck, A19L10) as a loading control. After washing with TBST, the membranes were incubated with horseradish peroxidase (HRP)-conjugated secondary antibodies (goat anti-rabbit IgG, 1:1000, Cell Signaling Technology, 7074) for 1 h at room temperature. Protein bands were visualized using enhanced chemiluminescence (ECL) substrate (Millipore, WBKLS0500) and imaged using a ChemiDoc imaging system (Bio-Rad). Band intensities were quantified using ImageJ, and target protein expression levels were normalized to GAPDH. All experiments were performed in triplicate.

### Immunofluorescence staining for NF-κB p65 nuclear translocation

Frozen left ventricular tissue sections were fixed with 4% paraformaldehyde for 15 min at room temperature, permeabilized with 0.2% Triton X-100 in PBS for 10 min, and blocked with 5% normal goat serum in PBS for 1 h at room temperature. The sections were then incubated overnight at 4 °C with a mixture of primary antibodies: mouse anti-NF-κB p65 (NF-κB Activation, Nuclear Translocation Assay Kit, 1:100, Beyotime, SN371) and rabbit anti-cardiac troponin T (cTnT, 1:200, Abcam, ab8295) to identify cardiomyocytes. After three washes with PBS, the sections were incubated with a mixture of secondary antibodies: Cy3-conjugated goat anti-mouse IgG (1:500, Beyotime, A0521) for NF-κB p65 and Alexa Fluor 488-conjugated goat anti-rabbit IgG (1:500, Beyotime, A0408) for cTnT, for 1 h at room temperature in the dark. Nuclei were counterstained with DAPI (1 μg/mL, Sigma-Aldrich, D9542) for 5 min. Fluorescence images were captured using a confocal microscope (Olympus FV3000). For each heart, 10 randomly selected fields were analyzed. Nuclear translocation of NF-κB p65 was quantified specifically in cTnT-positive cardiomyocytes by measuring the ratio of nuclear to cytoplasmic fluorescence intensity using ImageJ software. At least 100 cardiomyocytes per animal were analyzed. A blinded investigator performed all image acquisition and quantification procedures.

### Statistical analysis

Data were primarily presented as mean ± SEM of pooled results from both male and female rats, unless significant gender effects were observed, in which case, results were displayed separately in dot plots. Distinct symbols were used to identify each data point for each sex. The study was not designed to evaluate sex as a primary biological variable; therefore, all survival- and gender-based analyses are regarded as exploratory. Distributions were inspected using the Shapiro–Wilk test, and variance homogeneity was assessed using Levene’s test (GraphPad Prism 10). For normally distributed data with homogeneous variances, two-way ANOVA was performed with factors such as Treatment (Untreated, EMPA, PFD, and EMPA + PFD) and Sex. *Post hoc* comparisons were adjusted using Tukey’s method. If interaction terms were significant, simple main effects were analyzed within each sex using one-way ANOVA or unpaired t-tests. For data violating parametric assumptions, nonparametric factorial analyses (Aligned Rank Transform) were performed in Python (v3.13).

For body weight, data analyses were conducted using Python (version 3.13), leveraging the pandas library for data manipulation and descriptive statistics, scipy.stats for normality (Shapiro–Wilk) and homogeneity of variance (Levene’s) tests, statsmodels for linear mixed-effects modeling (mixed LM with restricted maximum-likelihood estimation), and pingouin for *post hoc* repeated-measures ANOVA, pairwise t-tests with Bonferroni correction, Bayes factors, and Hedges’ g effect sizes. All tests were two-sided with α = 0.05; no multiple-comparison adjustments were applied beyond Bonferroni’s correction for pairwise tests. Assumptions were largely met, with minor violations noted but not altering model convergence.

### Pharmacological synergy analysis (Bliss independence model)

To evaluate pharmacological synergy between EMPA and PFD, we applied the Bliss independence model, which compares the observed combinatorial effect of the dual therapy against a theoretically predicted additive effect. The fractional effect for a given drug (*E*_drug_) was calculated using the following formula:

$$E_{Drug}=\frac{{Mean}_{Untreated}-{Mean}_{Treated}}{{Mean}_{Untreated}-{Mean}_{Control}}.$$

Next, the theoretical expected additive fractional effect (E_Expected_) of the combination therapy was calculated using the Bliss independence equation:

$$E_{Expected}=E_{EMPA}+E_{PFD}-\left(E_{EMPA}\;x\;E_{PFD}\right).$$

Synergy was confirmed if the observed combinatorial benefit was mathematically greater than the predicted additive effect, whereas an observed benefit equal to or less than the prediction was classified as strictly additive or sub-additive, respectively.

## Results

### The combination of a high-fat diet and L-NAME-induced pathological characteristics of HFpEF in rats

For this experiment, rats were fed with HFD + L-NAME for 5 consecutive weeks to produce HFpEF characteristics. No significant change in body weight following HFD + L-NAME treatment was observed at baseline (day 7; p = 0.104), nor was there a sex effect (p = 0.924) (Supplementary Figure S1A). However, a significant main effect of time emerged, with body weight increasing significantly on day 21 (p = 0.044), day 28 (p < 0.001), and day 35 (p < 0.001) relative to day 7. Significant HFD + L-NAME treatment × time interactions on day 21 (p = 0.019), day 28 (p < 0.001), and day 35 (p = 0.001) indicated that HFpEF subjects exhibited a steeper body weight increase over time compared to controls. *Post hoc* T-tests confirmed significant induction differences at all time points. Within-group repeated-measures ANOVAs revealed significant time effects for both Control (p < 0.0001) and HFpEF (p < 0.0001), with pairwise comparisons (Bonferroni-corrected) showing progressive weight increases (HFpEF day 7 vs. day 28, p < 0.0001).

Rats fed with HFD + L-NAME also developed an increase in systolic and diastolic blood pressure (p < 0.0001, Supplementary Figure S1B). These changes coincided with glucose intolerance, as evidenced by a significant increase in fasting and postprandial blood glucose levels (Supplementary Figure S1C) after 5 weeks compared with rats on normal chow diet (p < 0.0001). HFD + L-NAME also elevated the total white blood cell count (p < 0.002) (Supplementary Figure S1D). Furthermore, rats fed with HFD + L-NAME also demonstrated a significantly increased neutrophil:lymphocyte ratio (NLR), indicating systemic inflammation (Supplementary Figure S1E). Notably, the significant increase in the platelet-to-lymphocyte ratio (PLR) was affected by sex, as analyzed using two-way ANOVA (p < 0.015). Tukey’s multiple comparisons test showed that the increase in PLR was significant between male chow-fed and L-NAME + HFD-fed rats (p = 0.0036), whereas no difference was observed in female rats between the two groups.

**FIGURE 1:**
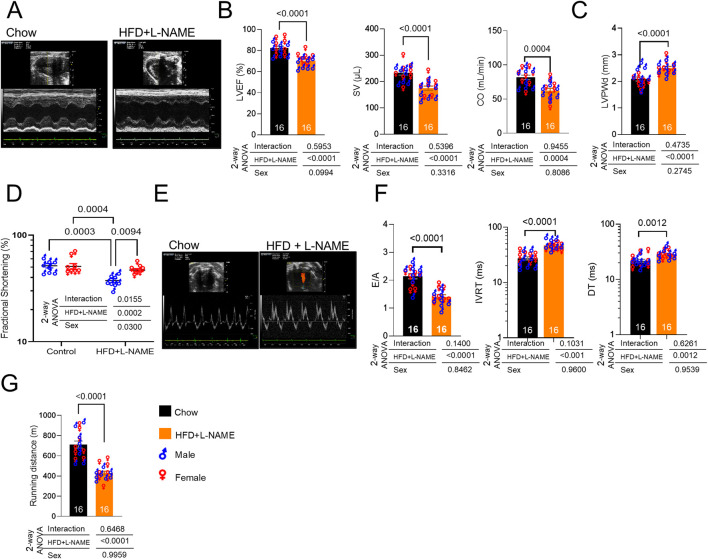
Induction of HFpEF in rats using a high-fat diet and L-NAME **(A)** Representative left ventricular M-mode echocardiographic images illustrating cardiac structure and function in control and HFpEF-induced rats following 5 weeks of L-NAME and high-fat diet administration. **(B)** Quantitative analysis of systolic function parameters, including left ventricular ejection fraction (LVEF), stroke volume (SV), and cardiac output (CO), demonstrating significant impairment in HFpEF rats (p < 0.005 versus Chow). **(C)** Increased left ventricle posterior wall diameter (LVPWd) observed in HFpEF rats. **(D)** Reduced fractional shortening (FS), predominantly in male rats subjected to HFD + L-NAME, indicating a sex-related difference (p < 0.003 versus control within male rats). **(E,F)** Diastolic function assessment via mitral Doppler echocardiography showing a significantly decreased E/A ratio, along with prolonged isovolumetric relaxation time (IVRT) and deceleration time (DT) in HFpEF rats (p < 0.005 versus chow). **(G)** Exercise tolerance, evaluated by treadmill running distance, is markedly reduced in HFpEF rats (p < 0.0001 versus Chow). Data are expressed as mean ± SEM, pooled from both sexes in the absence of sex-L-NAME interaction, as determined using two-way ANOVA with Tukey’s *post hoc* test; significance threshold set at p < 0.05.

To examine the heart function between the two groups, M-mode echocardiography was performed. From a parasternal long-axis view, impairment of left ventricular (LV) systolic function was evident in the HFD + L-NAME-treated group (Figure 1A). Heart function also worsened significantly, as shown by the decrease in LVEF (80%–70%, p < 0.0001), SV (230 µL–170 μL, p < 0.00011), CO (70 mL/min to 60 mL/min, p < 0.0004, Figure1B), and end-diastolic left ventricular posterior wall thickness (LVPWd, 2.09 mm–2.512 mm, Figure 1C). The decrease in fractional shortening was significantly lower in the male HFD + L-NAME-treated group as compared to the chow-treated groups, regardless of sex. It was also significantly lower compared to the female HFD + L-NAME-treated group (P < 0.009, Figure 1D).

Furthermore, mitral Doppler echocardiography (Figure 1D) was performed to confirm diastolic dysfunction, a hallmark of HFpEF. Compared to rats fed a standard chow diet (healthy control), HFD+L-NAME rats had a significantly lower E/A ratio (p < 0.0001), longer IVRT (p < 0.0001), and longer DT (p < 0.0012; Figures 1E,F). The reduced exercise tolerance of HFD+L-NAME rats, as measured by the running distance (Figure 1G), was consistent with their impaired heart function (p < 0.0001).

**FIGURE 2:**
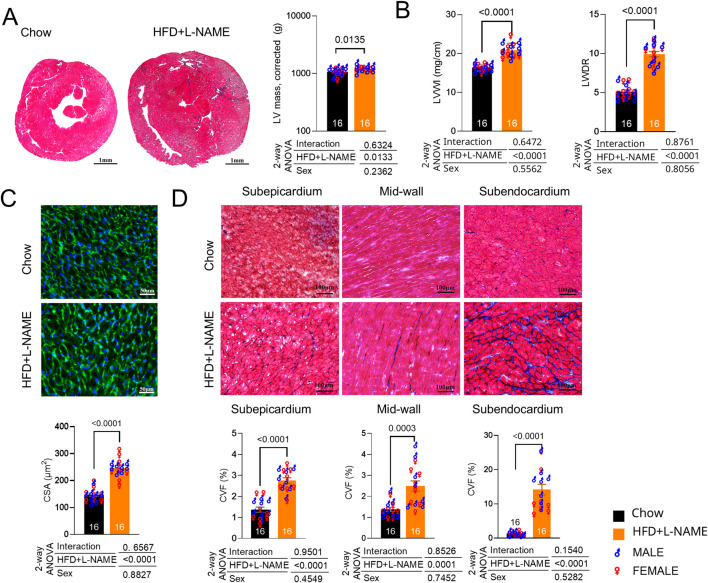
Structural and histological changes in HFpEF rats. **(A)** Representative Masson’s trichrome staining of mid-ventricular short-axis sections (scale bar = 1 mm) and quantification of left ventricular (LV) mass demonstrate increased myocardial fibrosis and LV mass in HFpEF rats (p = 0.0135 vs. Chow). **(B)** The left ventricle weight index (LVWI) and lung wet-to-dry weight ratio (LWDR) are also elevated. **(C)** Representative images of myocyte cross-sectional area (CSA) confirm cardiomyocyte hypertrophy following HFD + L-NAME administration (p < 0.0001 vs. chow). **(D)** Collagen volume fraction (CVF) analysis indicates increased fibrotic deposition in the subendocardium (p < 0.001 vs. chow), myocardium (p = 0.003 vs. chow), and subepicardium (p < 0.001 vs. chow) in HFpEF rats. All histological images are derived from heart sections of male rats. Data are presented as mean ± SEM, pooled from both sexes when no interaction between sex and L-NAME was observed, as determined using two-way ANOVA with Tukey’s *post hoc* test. Significance was set at p < 0.05.

Gross histology revealed hypertrophic hearts in the HFD + L-NAME group, as evident by a significant increase in left ventricular mass (p < 0.0135) (Figure 2A), LVWI (p < 0.0001), and LWDR (p < 0.0001) (Figure 2B). This was supported by cross-sectional views of the hearts of HFD + L-NAME rats, which showed enlarged myocytes (Figure 2C) and increased collagen deposition in the subendocardial (P < 0.0001), mid-wall (p = 0.0003), and subepicardial layers (p < 0.0001) (Figure 2D). For subsequent experiments, we used 5-week-old HFD + L-NAME rats as the disease model (hereafter referred to as HFpEF).

### Effects of co-treatment with EMPA and PFD on body weight and survival in HFpEF rats

**FIGURE 3:**
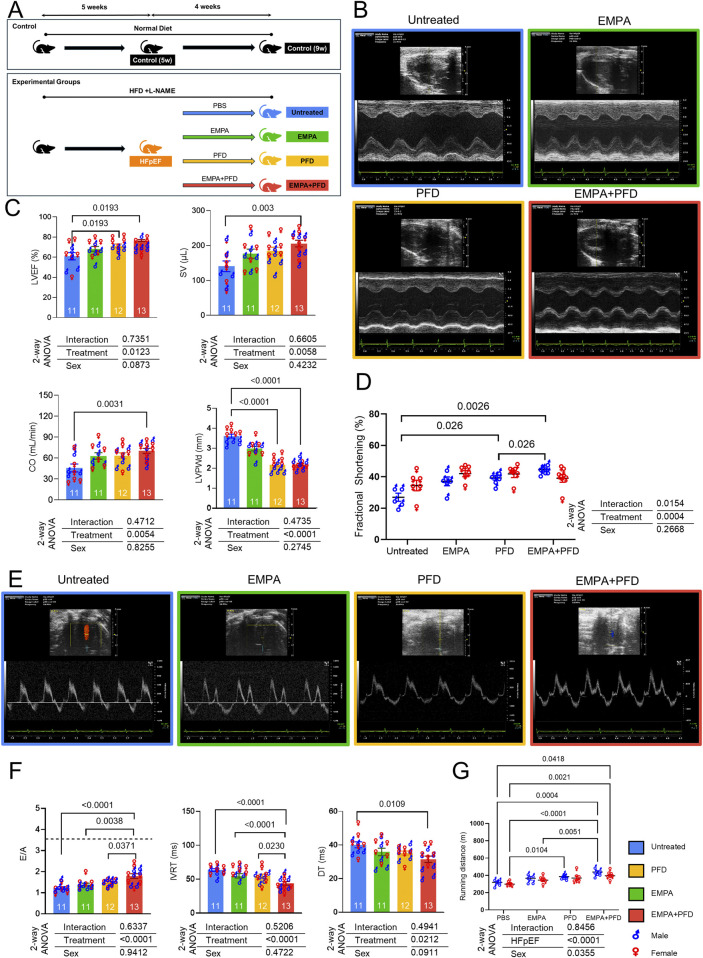
Impact of empagliflozin and pirfenidone on HFpEF outcomes **(A)** Study design schematic outlining HFpEF induction, treatment groups, and endpoints. **(B,C)** Representative echocardiographic M-mode images and quantification of LVEF, CO, and SV reveal significant improvements in EMPA + PFD-treated rats (p < 0.05 vs. untreated HFpEF), demonstrating improved cardiac systolic function in EMPA + PFD-treated rats compared to untreated HFpEF controls. **(D)** The benefit of EMPA + PFD in fractional shortening was only observed in male rats (P = 0.05 vs. untreated HFpEF). **(E,F)** Diastolic function indices, including E/A ratio, IVRT, and DT, are significantly improved following EMPA + PFD treatment (p < 0.05 vs. untreated HFpEF). **(G)** Exercise tolerance test results show a significant increase in running distance in EMPA + PFD-treated rats (p < 0.0001 vs. untreated HFpEF rats). Representative echocardiographic images presented in the figure were taken from male rats. Data are presented as mean ± SEM of the pooled dataset of both sexes; differences were analyzed using two-way ANOVA with Tukey’s *post hoc* test, with significance determined at p < 0.05. Iconography in this figure was sourced from Microsoft 365.

Figure 3A presents the overall experimental design. The chemical structures of EMPA and PFD used in this study are shown in Supplementary Figure S2A. Kaplan–Meier analysis showed that median survival times were undefined for EMPA + PFD in both genders, indicating >50% survival at 28 days (Supplementary Figure S2B). However, the Cox model, with the untreated group as the reference, showed hazard ratios of 0.99 (p = 0.99) for EMPA, 0.87 (p = 0.83) for PFD, and 0.57 (p = 0.44) for the EMPA + PFD group, with all confidence intervals including 1, indicating no significant treatment effects. Additionally, log-rank tests comparing each treatment group to the untreated control yielded P-values of 0.9934, 0.8137, and 0.4383, respectively, confirming no differences in survival distributions. Due to the study’s low power (only 17 events and 73% censoring) and poor model fit (concordance = 0.55), the trend is inconclusive.

Next, we compared EMPA, PFD, and EMPA + PFD over the next 4 weeks with the untreated control that received only PBS. For comparison, healthy rats on a normal chow diet served as controls at weeks 5 (control 5w) and 9 (control 9w). Time-lapse changes in the rat body weights in response to treatments on days 0, 7, 14, 21, and 28 were recorded (Supplementary Figure S2C). Linear mixed-effects modeling revealed a significant interaction between treatment and time for body weight (p < 0.001), with no main effects of treatment or gender. In the control group, body weight increased progressively over 28 days (p < 0.001), reaching means of 553.14 ± 23.66 g (female rats) and 559.50 ± 18.23 g (male rats) at day 28 (Supplementary Figure S2D). Similarly, the PFD-treated group exhibited substantial weight gain (p < 0.001), culminating in 584.00 ± 26.24 g (female rats) and 575.60 ± 21.30 g (male rats) at day 28, with *post hoc* comparisons indicating significant increases relative to baseline (p < 0.001). In contrast, EMPA monotherapy attenuated weight gain, resulting in stable or modestly reduced weights (male: 503.88 ± 16.81 g on day 0 to 472.40 ± 25.80 g on day 28; no overall time effect, p = 0.31), while the combination therapy (EMPA + PFD) induced the most pronounced reductions (p < 0.0001; day 0 vs. day 28, p = 0.006), yielding endpoint means of 449.50 ± 34.90 g (female rats) and 480.29 ± 29.02 g (male rats). *Post hoc* analyses on day 28 confirmed significant differences between EMPA + PFD and control (p < 0.001), between EMPA and control (p < 0.001), and PFD versus both active treatments (all p < 0.001), underscoring the weight-lowering efficacy of EMPA-containing regimens (Supplementary Figure S2E).

### EMPA and PFD co-treatment enhances cardiac function and reduces hypertrophy and fibrosis in HFpEF rats

To evaluate the effects of the EMPA + PFD combination on heart function and structure, echocardiographic and histological analyses were performed. EMPA + PFD treatment consistently improved LVEF (75.6% ± 0.9% vs. 60.9 ± 3.64, P < 0.0193), SV (205.6 ± 9.4 µL vs. 140.5 ± 15.4 µL, P = 0.003), and CO (70.4 ± 3.6 mL/min vs. 445.8 ± 5.6 mL/min, P = 0.0031), compared to the untreated control (Figures 3B,C). Two-way ANOVA on ranks for fractional shortening on HFpEF rats revealed a significant main effect of treatment (p = 0.0004) and a treatment × sex interaction (p = 0.015), but no main effect of sex (p = 0.267) (Figure 3D). In male rats, Kruskal–Wallis tests indicated significant treatment effects (p = 0.0014), while *post hoc* Mann–Whitney U tests revealed differences between untreated and PFD (adjusted p = 0.026), untreated and EMPA + PFD (adjusted p = 0.026), and PFD and EMPA + PFD (adjusted p = 0.026). In contrast, untreated vs. EMPA comparison was not significant after adjustment (adjusted p = 0.191). No treatment effects were observed in female rats (p = 0.134). These results suggest that PFD and EMPA + PFD significantly modulate the response variable in male rats, but not in female rats, highlighting sex-specific treatment responses.

Doppler echocardiographic analysis revealed that EMPA + PFD significantly improved the EA ratio of HFpEF rats (1.79 ± 0.11), and the effect was superior to that of untreated (1.22 ± 0.06, p < 0.0001), EMPA (1.39 ± 0.07, p = 0.0038), and PFD (1.488 ± 0.04, p = 0.0371) alone treatment, analyzed using two-way ANOVA (Figures 3E,F). Similarly, the combination showed a significant reduction in IVRT (p < 0.0001 vs. untreated; p < 0.0001 vs. EMPA; p = 0.023 vs. PFD) and DT (p = 0.0109 vs. untreated). The EMPA + PFD combination showed superior exercise tolerance, measured by running distance (414.6 ± 12.51 m), compared to EMPA alone (354.1 ± 12.9 m, P < 0.0001) and the untreated group (307.5 ± 8.68 m, P < 0.0001, Figure 3G).

**FIGURE 4:**
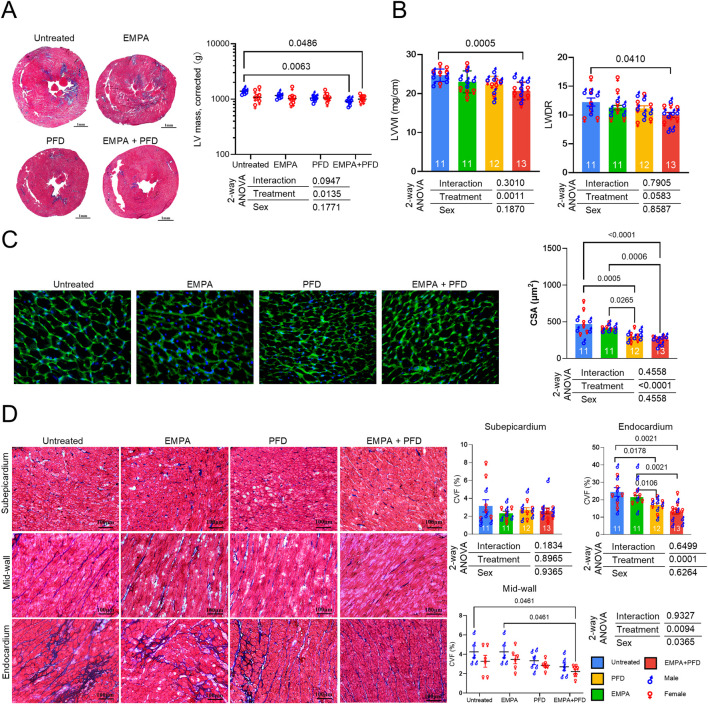
Histological evidence of reduced hypertrophy and fibrosis with EMPA + PFD treatment. **(A)** Masson’s trichrome staining shows reduced myocardial fibrosis, and left ventricular mass is significantly lower in EMPA + PFD-treated rats compared to HFpEF controls (p < 0.05). **(B)** When compared to control, left ventricle weight index (LVWI) was significantly lower in EMPA + PFD (adjusted p < 0.001), while difference in the lung wet-to-dry weight (LWDR) ratio was modest (adjusted p < 0.05). **(C)** Myocyte cross-sectional area (CSA) measured using WGA indicates reduced hypertrophy in EMPA + PFD-treated rats compared to HFpEF controls (p < 0.001). **(D)** Collagen volume fraction (CVF) analysis shows that EMPA + PFD treatment significantly reduces fibrosis, particularly in the mid-wall (p = 0.0267 vs. untreated HFpEF) and endocardium (p = 0.0021 vs. untreated HFpEF), with no significant changes in the subepicardial region. All histology images are presented using heart sections from male rats. Data are expressed as mean ± SEM, with two-way and Tukey’s *post hoc* tests used for analysis.

Upon histological examination of the heart’s short-axis cross-section, untreated rats displayed the most pronounced hypertrophic remodeling (Figure 4A). HFpEF rats treated with EMPA + PFD exhibited a notable reduction in LV mass, but this was limited to male HFpEF rats (p = 0.0063). Furthermore, the EMPA + PFD treatment regimen resulted in a significant decrease in LVWI (p = 0.0005 vs. untreated) and LWDR (p = 0.0410 vs. untreated), suggesting diminished pulmonary congestion (Figure 4B). Analysis also showed a significant reduction in CSA in the left ventricular myocardium when treated with PFD (330.8 ± 10.6 µm^2^ vs. untreated 421.8 ± 21.3 µm^2^, p = 0.0012) and EMPA + PFD (282.3 ± 6.95 µm^2^ vs. untreated 421.8 ± 21.3 µm2, p = 0.0006) (Figure 4C).

The dual therapy also effectively reduced cardiac fibrosis in the endocardium (13.21% ± 1.45% vs. untreated 24.47% ± 2.51%, p < 0.0237) and mid-wall (2.45% ± 0.2% vs. untreated 3.73% ± 0.44%, p = 0.0184; Figure 4D), and the effect was greater than that of EMPA-alone treatment (3.31% ± 0.22%, p = 0.0157). PFD also significantly reduced cardiac fibrosis in the endocardium (16.88% ± 1.12%) compared with the untreated control (p = 0.0178), and the effect was greater than that of EMPA alone (p = 0.0105)—no difference was observed when compared to EMPA + PFD. Notably, no discernible changes in fibrosis extent were observed in the subepicardial layer among the treatment groups.

Although no changes in systolic and diastolic blood pressure were observed in response to any treatment (Supplementary Figure S3A), the EMPA-only and EMPA + PFD-treated cohorts showed significantly lower fasting and postprandial blood glucose levels (both p < 0.0001 versus untreated; Supplementary Figure S3B), as expected. These changes were not observed in the PFD-only treatment groups, and none of the treatments influenced the WBC count or PLR. Interestingly, NLR was significantly lower in the EMPA + PFD group (p < 0.0325 versus control; Supplementary Figure S3C). No gender effect was observed, as analyzed using two-way ANOVA.

### RNA sequencing reveals transcriptional changes and distinct gene expression profiles in empagliflozin and pirfenidone-co-treated HFpEF rats

**FIGURE 5:**
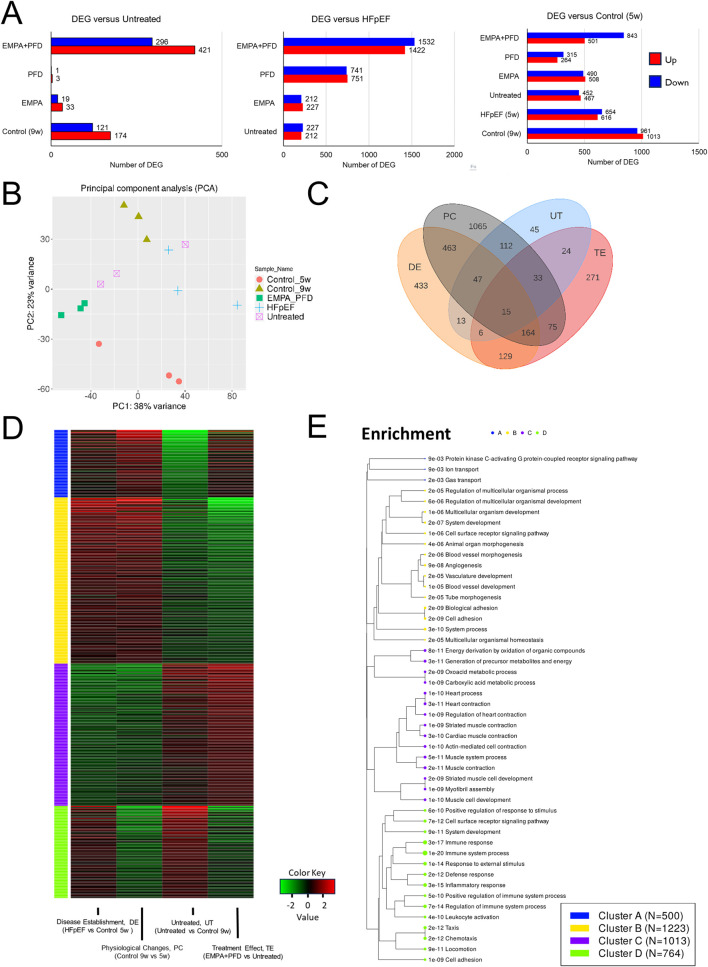
RNA sequencing reveals distinct gene expression profiles in EMPA + PFD-treated HFpEF male rats. **(A)** Differential expression analysis comparisons (adjusted p-value < 0.05 and absolute fold change >2). Bar chart presenting the number of upregulated and downregulated DEGs for EMPA + PFD, PFD, EMPA, and control (9w) vs. untreated (left) or vs. HFpEF (middle), highlighting significant transcriptional shifts. **(B)** Principal component analysis (PCA) demonstrating clustering of gene expression profiles, with EMPA + PFD-treated rats showing a shift toward the healthy control (5w) phenotype. **(C)** Venn diagram illustrating the overlap of DEGs between disease establishment (DE), physiological changes (PC), untreated (UT), and treatment effect (TE) comparisons. **(D)** Heatmap of hierarchical clustering of DEGs across study groups, illustrating upregulation and downregulation patterns. **(E)** K-means clustering analysis of enriched Gene Ontology (GO) biological processes, with pathways associated with protein kinase C-activating G-protein-coupled receptor, inflammation, and metabolism showing significant modulation in EMPA + PFD-treated rats. Data were analyzed using DESeq2, with an FDR cut-off of 0.05 and a minimum fold-change of 2.

We sequenced ventricular RNA (n = 3 male hearts per group) to examine the underlying transcriptional changes in each group. (note: two samples, one from the EMPA and one from the PFD monotherapy groups, were excluded due to corrupted data files. Total read counts, data distribution, and sample correlations are presented in Supplementary Figure S4). Strikingly, DEG analysis revealed that although individual monotherapies induced minimal transcriptional shifts compared to the untreated HFpEF group (52 DEGs for EMPA alone; four DEGs for PFD alone), the combination therapy triggered a massive transcriptomic reprogramming, yielding 717 DEGs (421 upregulated and 296 downregulated) (Figure 5A).

To contextualize these changes, we tracked disease progression across our developmental timeline. Compared to the 9-week untreated HFpEF group, the healthy 9-week controls yielded 295 DEGs, representing the genetic signature of unchecked disease progression. When compared with the 5-week pretreatment baseline, the EMPA + PFD group exhibited the highest overall variance (2,954 DEGs), vastly outperforming the transcriptomic shifts observed with PFD (1,492 DEGs), EMPA (439 DEGs), or untreated progression (439 DEGs). Collectively, these developmental and comparative data emphasize that dual therapy does not merely stall pathology but actively and uniquely reshapes the cardiac transcriptome (Figure 5A).

The principal component analysis (PCA) captures 61% of the total variance in its first two components and reveals three distinct biological axes driven by therapeutic intervention, disease state, and time. The most prominent driver of variance (PC1, 38%) highlights the profound impact of EMPA + PFD treatment. The treated samples form a tightly clustered group that completely separates from the disease model (HFpEF) along the major axis. Moreover, the treatment does not simply revert the samples to a baseline healthy state. Instead, the intervention powerfully overrides the disease’s chaotic variability, forcing the myocardium into a highly uniform, newly compensated “treatment phenotype” (Figure 5B).

We identified four DEG groups to examine meaningful events, namely, the heart physiology changes (control 9w vs. 5w, PC), the HFpEF disease establishment (HFpEF vs. control 5w, DE), the untreated consequence (untreated vs. control 9w, UT), and the effects of EMPA + PFD treatment (EMPA + PFD vs. untreated, TE). Using a Venn diagram to delineate the involvement of the DEGs in each event, we found that 314 genes in TE (43.7% of the total DEGs in TE) overlapped with DE, of which 179 genes (57% of the DEGs of TE∩DE) were the intersection of PC and 129 genes (41% of the DEGs of TE∩DE) were shared between DE and TE, indicating targeted reversal of the primary disease pathways (Figure 5C). Gene Ontology (GO) term analysis describing the biological processes of individual comparisons is shown in Supplementary Figure S5. Interestingly, in TE, upregulated DEGs were primarily related to metabolic events and cardiac structure, while the downregulated DEGs were mostly related to inflammatory responses.

To compare the expression patterns of DEGs across the four events, K-means clustering was performed, and the results are presented as heatmaps (Figures 5D,E). Among the four enrichment clusters, the TE group showed reversed gene expression patterns in clusters A (blue) and D (green) compared to the UT condition. These DEGs in cluster A were enriched for protein kinase C (PKC)-activating G-protein-coupled receptor signaling and ion/gas transport. In contrast, DEGs in cluster D were associated with immune/inflammatory responses. In contrast, DEGs in clusters B (yellow) and C (purple) showed no differences between the UT and TE conditions, indicating minimal EMPA + PFD effects on these two gene sets.

**FIGURE 6:**
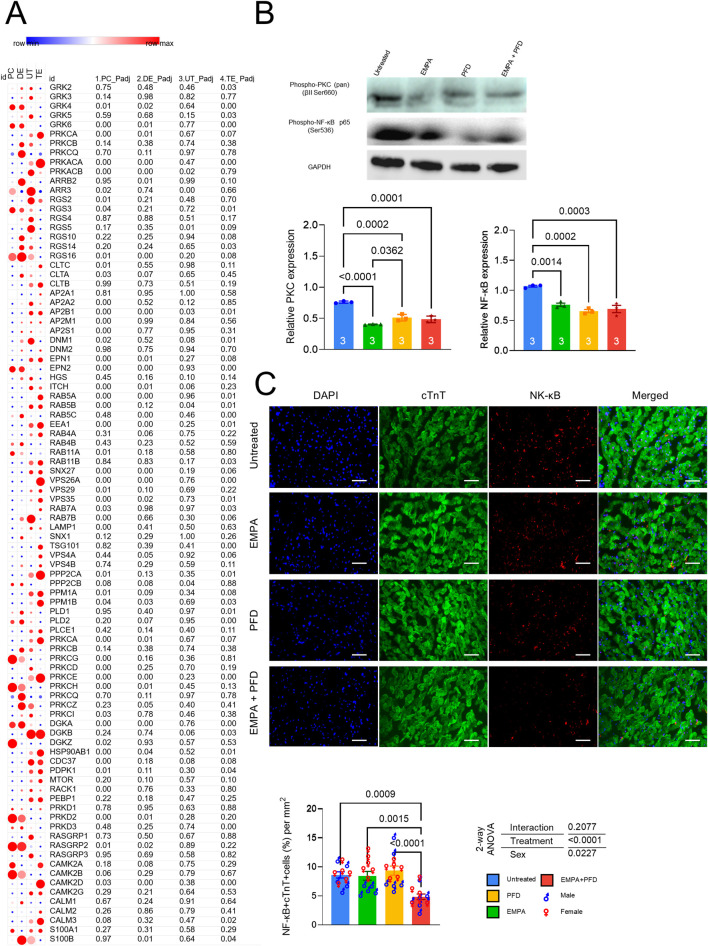
PKC–GPCR signaling pathway dysregulation drives NF-κB-mediated myocardial inflammation in HFpEF and is attenuated by EMPA + PFD. **(A)** Overview of DEGs of PC, DE, UT, and TE specific to the PKC–GPCR signaling pathway. Bubble plot displaying Log_2_FC with corresponding adjusted p-values (Padj) for each gene across four experimental comparisons: physiological cardiac maturation (PC: healthy 9-week vs. healthy 5-week rats), disease establishment (DE: HFpEF vs. healthy rats at 5 weeks), untreated chronic disease (UT: HFpEF vs. healthy rats at 9 weeks), and treatment effect (TE: treated vs. untreated HFpEF rats at 9 weeks). **(B)** Western blot analysis of phospho-PKC (Pan/βII-Ser660) and phospho-NF-κB p65 (Ser536) with GAPDH as a loading control. Semiquantitative analysis of the respective proteins was normalized to GAPDH expression (n = 3). Differences were analyzed using one-way ANOVA followed by Tukey’s *post-hoc* test. **(C)** Immunofluorescence staining of heart sections demonstrating nuclear translocation of NF-κB in cardiac troponin T-positive cardiomyocytes. Statistical significance was determined using ANOVA with *post-hoc* testing, with p < 0.05 considered significant.

We analyzed the gene set specific to PKC-activating GPCR signaling by comparing DEGs across the PC, DE, UT, and TE groups (Figure 6A). EMPA + PFD produced a broad and directionally coherent transcriptional response across the PKC–CaMKII–calcium–lipid signaling network. Among the most significantly upregulated genes, PRKCE (log_2_FC = +1.54, P_adj_ < 0.001) and CAMK2D (log_2_FC = +0.93, P_adj_ < 0.001) showed the strongest treatment-driven recovery, both having been markedly downregulated at disease onset, indicating that treatment restores calcium-dependent kinase signaling suppressed during HFpEF establishment. PRKACA, a catalytic subunit of PKA, was similarly upregulated (log_2_FC = +1.12, P_adj_ = 0.002), alongside the chaperones HSP90AB1 (log_2_FC = +0.72, Padj = 0.008) and PPP2CA (log_2_FC = +0.97, P_adj_ = 0.009), suggesting restoration of kinase proteostasis and phosphatase activity. CALM3 was significantly upregulated (log_2_FC = +0.71, P_adj_ = 0.023), pointing to recovery of calmodulin-mediated calcium sensing. VPS26A (log_2_FC = +0.91, P_adj_ < 0.001) and several endosomal trafficking components, including RAB5A, RAB5B, EEA1,VPS35, and AP2B1, were also upregulated, indicating treatment-associated restoration of intracellular vesicle trafficking and receptor recycling.

EMPA + PFD treatment also significantly reduced genes involved in lipid signaling and inflammation. S100B showed the greatest decrease (log_2_FC = −2.49, P_adj_ = 0.036), reversing its increase at disease onset and indicating reduced advanced glycation end product (RAGE)-driven inflammation. Diacylglycerol Kinase Alpha expression was also downregulated (log_2_FC = −0.77, P_adj_ = 0.001), suggesting normalization of DAG metabolism. Similarly, PLD1 and PLD2 levels were suppressed, indicating decreased phospholipase D activity. Genes related to GPCR desensitization, GRK4 and GRK6, were downregulated, implying improved receptor responsiveness. RGS3 and RGS14, involved in G-protein regulation, were also downregulated. Additionally, PRKD3 and EPN2 showed reduced expression, indicating broad remodeling of kinase and endocytic signaling pathways.

Next, we sought to determine whether this translated into altered protein activation. Western blotting revealed robust upregulation of phospho-PKC (pan) and its downstream effector, phospho-NFκB p65, in the untreated group. Treatment with EMPA, PFD, or the combination therapy effectively suppressed this signaling axis (Figure 6B). To define the spatial context of this response, immunofluorescence on myocardial sections was performed, revealing that the treatment-induced decrease in NFκB activity was characterized by loss of nuclear p65 localization in cardiomyocytes. Together, these results demonstrate that the therapeutic efficacy of EMPA and PFD is linked to inhibition of a convergent PKC–NF-κB inflammatory circuit (Figure 6C).

### Bliss analysis unveils targeted myocardial synergy

To quantitatively evaluate whether the combinatorial efficacy of EMPA and PFD was synergistic or strictly additive, we applied the Bliss independence model across all systemic, structural, and functional endpoints (Table 1). Strikingly, the Bliss analysis revealed a highly compartmentalized pattern of synergy that was uncoupled from systemic hemodynamic unloading. Systemic parameters, including systolic blood pressure (SBP), diastolic blood pressure (DBP), LWDR, and markers of systemic inflammation and thrombosis (NLR and PLR), showed strictly additive or sub-additive improvements. In contrast, the combination therapy synergistically reversed pathological left ventricular hypertrophy, reducing corrected LV mass, coupled with reversal of cardiomyocyte enlargement (CSA) and localized clearance of deep myocardial fibrosis (endocardial CVF). The combination therapy also synergistically improved diastolic compliance, increasing the E/A ratio from 1.14 (untreated) to 1.86, exceeding the Bliss analysis-expected value of 1.64. Corresponding synergistic improvements were observed in systolic mechanics, including LVEF and SV.

**TABLE 1:** Bliss independence analysis of empagliflozin and pirfenidone dual therapy in a two-hit HFpEF model.

Parameter	Control (healthy)	Untreated (HFpEF)	EMPA monotherapy	PFD monotherapy	Expected Bliss	EMPA + PFD (combo)	Synergy status
Systemic/Circulatory							
Systolic BP (mmHg)	111.06	148.5	142.38	143.19	137.93	142.19	Sub-additive
Diastolic BP (mmHg)	76.31	118.69	112.62	111.38	106.36	113.12	Sub-additive
Lung wet-to-dry weight ratio	5.19	12.26	11.29	11.12	10.3	10.44	Sub-additive
NLR (inflammation)	0.7	2.93	2.7	2.51	2.32	2.39	Sub-additive
PLR (thrombosis)	139.61	290.3	281.54	299.28	281.54	284.02	Sub-additive
Body weight (g)	472.09	580.18	478.73	517.08	474.85	466.08	Synergistic
Running distance (m)	646.83	307.55	354.13	377.32	414.32	414.57	Perfectly additive
Tissue fibrosis							
Endo CVF (%)	1.62	24.56	21.18	17.27	14.96	13.84	Synergistic
Mid-wall CVF (%)	1.55	3.79	3.24	3.14	2.75	2.75	Perfectly additive
Epi CVF (%)	1.54	2.79	2.47	2.63	2.35	2.47	Sub-additive
Cardiac structure							
LV Mass corr. (mg)	1055.9	1242.16	1106.81	1059.55	1056.9	967.47	Synergistic
LVPWd (mm)	2.46	2.45	2.61	2.54	2.46	2.82	Synergistic
LVEDd (mm)	6.91	9.01	8.12	7.59	7.3	7.31	Perfectly additive
Myocyte CSA (µm^2^)	260.3	466.54	413.96	305.02	293.62	257.67	Synergistic
Cardiac function							
LVEF (%)	82.61	60.91	68.31	71.38	75.21	76.59	Synergistic
SV (μL)	232.36	140.5	176.61	184.99	203.61	205.6	Synergistic
LVEDV (μL)	287.73	202.91	241.24	242.03	262.68	263.61	Synergistic
LVESV (μL)	55.37	62.41	64.63	58.71	58.71	58.32	Synergistic
E/A ratio	2.14	1.14	1.25	1.58	1.64	1.86	Synergistic
Fractional shortening (%)	53.03	34.61	39.78	40.52	44.03	41.63	Sub-additive
Cardiac output (mL/min)	82.69	45.78	63	63.41	72.4	70.39	Sub-additive
Heart rate (bpm)	356	329.82	357.36	341.58	356	349.92	Sub-additive
IVRT (ms)	28.16	63.15	50.03	45.93	39.26	39.55	Perfectly additive
Protein expression							
Phospho-PKC (a.u.)	0.5	1.35	0.95	0.8	0.66	0.55	Synergistic
Phospho-NF-κB p65 (a.u.)	0.45	1.25	0.90	0.8	0.65	0.55	Synergistic

Abbreviations: BP, blood pressure (systolic/diastolic); CO, cardiac output; CVF, collagen volume fraction (endo, endocardium; epi, epicardium); HFpEF, heart failure with preserved ejection fraction; EMPA, empagliflozin; PFD, pirfenidone; NLR, neutrophil-to-lymphocyte ratio; PLR, platelet-to-lymphocyte ratio; LV Mass Corr., corrected left ventricular mass; LVPWd, left ventricular posterior wall thickness at diastole; LVEDd, left ventricular end-diastolic internal diameter; LVEF, left ventricular ejection fraction; SV, stroke volume; LVEDV, left ventricular end-diastolic volume; LVESV, left ventricular end-systolic volume; E/A ratio, ratio of early (E) to late (A) ventricular filling velocity; IVRT, isovolumic relaxation time; PKC, protein kinase C; NF-κB, nuclear factor kappa-light-chain-enhancer of activated B cells; A.u., arbitrary unit.

Other parameters of which the effects were sub-additive are not listed here.

Moreover, quantitative densitometry and Bliss independence analysis confirmed that EMPA + PFD synergistically suppressed the PKC and NF-κB signaling cascade. Specifically, dual therapy reduced p-PKC activation significantly beyond the predicted additive effect (observed ratio: 0.55 vs. expected: 0.66), nearly restoring expression to healthy control levels (0.50). A parallel synergistic reduction was observed for p-p65 (observed ratio: 0.55 vs. expected: 0.65). Immunofluorescence further revealed that this systemic decrease in NFκB activity was driven by the loss of nuclear p65 localization specifically within cardiomyocytes. Collectively, these data suggest that the efficacy of the EMPA + PFD combination is mechanistically rooted in the synergistic inhibition of a convergent, profibrotic PKC/NF-κB circuit. Taken together, these data suggest that although EMPA and PFD act additively on systemic hemodynamics, their co-administration alters the local myocardial microenvironment, thereby synergistically halting and reversing HFpEF pathogenesis.

## Discussions

HFpEF continues to pose a formidable clinical challenge, with few interventions offering robust improvements in both morbidity and mortality. Despite recent evidence showing that SGLT2i can reduce hospitalization and cardiovascular mortality in patients with ejection fractions greater than 40% (Nassif et al., 2021; Spertus et al., 2022), their benefit in reversing diastolic dysfunction and structural remodeling in HFpEF remains modest (Anker et al., 2021; Yang et al., 2022). Our findings suggest that the antifibrotic drug PFD can augment the favorable effects of EMPA, enhancing cardiac function and mitigating pathological remodeling in a two-hit HFpEF rat model.

### The two-hit HFpEF model

We employed a two-hit model that combines L-NAME-induced endothelial dysfunction with a HFD to recapitulate key clinical features of HFpEF, including systemic inflammation, metabolic stress, and myocardial stiffness. This approach was informed by prior studies demonstrating that L-NAME/HFD suppresses nitric oxide (NO) bioavailability, promotes s-nitrosylation of inositol-requiring protein 1α (IRE1α), and impairs the IRE1α–XBP1 stress-adaptive pathway—mechanisms closely mirrored in human HFpEF myocardium (Schiattarella et al., 2019). Although some HFpEF models utilize genetic modifications or alternative stressors, the HFD + L-NAME combination effectively induced systolic and diastolic dysfunction in our rats, with LVEF remaining in the preserved range (>50%). We also report that 9 weeks of HFpEF induction is associated with a mortality of ∼30%, reflecting the severity of the two-hit model. Although similar two-hit models (6–8 weeks, 40%–45% HFD, 30–50 mg/kg/day L-NAME) also showed 0%–15% mortality from hypertensive crisis or renal failure (Li et al., 2023), the prolonged 9-week induction with a high L-NAME dose likely amplified cardiovascular strain and accelerated hypertension. Nonetheless, we recognized that our “two-hits” model did not account for aging, a key factor in the pathophysiology of clinical HFpEF. Future studies should incorporate additional stressors such as obesity, female predominance, volume overload, aging, or uninephrectomy to better replicate human HFpEF and improve the accuracy and translatability of therapeutic outcomes.

### Dosage considerations and synergy of dual EMPA–PFD therapy

In our study, we administered EMPA at a dose of 350 mg/kg/day to HFpEF rats, a level higher than typical human-equivalent exposures but well within the established preclinical safety margins. Preclinical data demonstrate that EMPA exhibits dose-dependent pharmacological effects, including glucosuria, polyuria, and body weight reduction, as well as adaptive changes such as renal tubular dilatation and mineralization (Bogdanffy et al., 2014). In repeat-dose rat studies, doses up to 300 mg/kg/day were associated with desirable pharmacodynamic outcomes, minimal target-organ effects, and no impact on survival (Bogdanffy et al., 2014). These studies established a no-observed-adverse-effect level of 100 mg/kg/day, while separate data confirmed tolerability at higher exposures approximating 26–45-fold the maximum recommended human dose (Boehringer Ingelheim Pharmaceuticals and Inc., 2026). Adverse effects, including moribund states, dehydration, and exacerbated multi-organ mineralization (e.g., in the kidney, liver, and heart), were observed only at supratherapeutic doses of 700 mg/kg/day in rats (>70-fold human exposure), with more severe renal tubular injury confined to 1,000 mg/kg/day in mice (Knight et al., 2018). Hence, by selecting this elevated dose, we aimed to exclude potential dose-dependent limitations of EMPA monotherapy that might preclude the observation of the true beneficial effects of pirfenidone in our HFpEF model, thereby isolating and amplifying the benefits of combined EMPA + PFD therapy.

A vast body of literature has suggested a direct effect of EMPA on the heart, conferring functional improvement in failing hearts through inhibition of NHE-1 and voltage-gated Na^+^ channels (Nav1.5), suppression of reactive oxygen species production, nucleotide-binding oligomerization domain-like receptor P3 (NLRP3) inflammasome activation, and proinflammatory cytokines (see review) (Zelniker and Braunwald, 2020). However, increasing skepticism with evidence demonstrated the lack of SLGT2 expression in the heart (Mourad et al., 2025; Mustroph et al., 2018; Anker and Butler, 2018), suggesting that the observed beneficial effect of EMPA was likely derived from either off-target actions (Dyck et al., 2022) or via indirect mechanisms (Chen et al., 2024; Wang et al., 2025), especially when EMPA has a higher affinity for SLGT2, up to 2,500-fold (Grempler et al., 2012). Although we observed a trend toward improvement in systolic and diastolic function in EMPA-treated rats, the difference was not statistically significant, despite improved glucose tolerance, weight loss, and exercise capacity. This does not contradict EMPA’s established cardiac benefits in other studies but may be attributable to factors such as the shorter treatment duration, choice of animal model, and subject species in this study. The “two-hit” preclinical model has mostly utilized mice, particularly C57BL/6 strains, in past studies (van Ham et al., 2022). In contrast, rat-based investigations are scarce, with only one study in Wistar rats involving 8 weeks of HFD/fructose plus L-NAME induction, followed by 8 weeks of EMPA treatment at 10 mg/kg/day (Author anonymous, 2024). Comparatively, mouse models show faster EMPA benefits, with improvements in diastolic function emerging as early as 3 weeks and by 8–9 weeks of oral dosing (Ranjbarvaziri et al., 2024; Liu et al., 2025).

Notably, we also identified a novel paradigm of compartmentalized synergy. Although the combination therapy elicited strictly additive or sub-additive improvements in systemic hemodynamics and fluid status, it synergistically reversed pathological cellular hypertrophy, cleared deep myocardial fibrosis, and, to a certain extent, restored diastolic compliance. These findings suggest that simultaneously targeting the SGLT2 metabolic axis and the TGFβ profibrotic axis uniquely optimizes the local myocardial microenvironment, achieving structural and functional rescue that is unattainable with either monotherapy alone. Because EMPA drives significant osmotic diuresis and natriuresis, reductions in cardiac mass and improved filling pressures are often attributed merely to decreased systemic preload, especially when a supratherapeutic dose was used in this study. However, our Bliss independence analysis robustly challenges this view. If the benefits of the EMPA + PFD combination were driven predominantly by exaggerated volume depletion, we would expect systemic and circulatory markers to exhibit the greatest combinatorial synergy. In contrast, systemic blood pressure (SBP/DBP), pulmonary congestion (LWDR), and markers of systemic inflammation (NLR/PLR) demonstrated strictly additive or sub-additive effects. Therefore, the profound synergy observed specifically within the myocardium cannot be dismissed as an artifact of massive systemic fluid loss, but rather represents a genuine, localized pharmacological interaction.

### Mechanistic considerations: linking fibrosis reduction to functional improvement

In the heart, EMPA prevents fibrosis in preclinical models by inhibiting the NHE1–NO pathway (Chung et al., 2023), along with reducing NCX upregulation, oxidative/nitrosative stress, and CaMKII activation—effects persisting in SGLT2-knockout mice, confirming SGLT2 independence (Mustroph et al., 2018; Lee et al., 2019; Schauer et al., 2024). Thus, EMPA’s antifibrotic actions are largely off-target as fibrosis attenuation persists in SGLT2-knockout models (Chen et al., 2024). In this study, PFD monotherapy outperformed EMPA alone in reducing fibrosis. At the same time, their combination significantly improved LV systolic/diastolic function, reduced pulmonary congestion, and enhanced exercise capacity within 4 weeks, suggesting a perfectly additive effect via EMPA’s metabolic/oxidative modulation and PFD’s direct TGF-β suppression (Sanders-van Wijk et al., 2020; Schiattarella et al., 2021).

Although EMPA has shown antifibrotic and diastolic benefits in longer-term preclinical models (e.g., 6–12 weeks at 10–30 mg/kg/day in ZSF1 rats), our 4-week study at a supratherapeutic dose (∼135-fold the human MRHD) revealed no significant improvement in cardiac function or fibrosis with monotherapy. This is consistent with clinical trials (EMPEROR-preserved, DELIVER, EMPA-REG OUTCOME, and EMPA-HEART 2 CardioLINK-7) showing reduced Hospitalization for heart failure without echocardiographic changes (Anker et al., 2021; Solomon et al., 2022; Rau et al., 2021; Connelly et al., 2023) and aligns with the findings of Pabel et al. (2018), who noted that acute functional effects are unlikely due to fibrosis regression. The short duration likely favored hemodynamic over structural remodeling. Notably, the combination with PFD accelerated antifibrotic effects, suggesting that targeting TGF-β signaling may overcome the temporal constraints of SGLT2 inhibition alone. Although SGLT2 inhibitors have also been reported to exert indirect antifibrotic effects in certain contexts (Yang et al., 2022; Lee et al., 2022; Daud et al., 2021; Tian et al., 2021), the more robust remodeling observed here indicates that PFD substantially amplifies or complements these benefits (Du et al., 2017).

### Transcriptional remodeling and fibrosis–inflammation axis

The comprehensive transcriptomic profiling reveals that the EMPA + PFD combination therapy exerts a powerful, synergistic effect on the failing myocardium, driving a transcriptional response that far exceeds that of either monotherapy. This massive gene regulation forces the treated tissues out of the heterogeneous HFpEF disease state and into a tightly clustered, highly distinct principal component space. Additionally, our PCA demonstrates that the intervention does not merely revert the heart to a naive physiological baseline. Instead, it establishes a novel, compensatory therapeutic phenotype while actively overriding the core pathogenic networks responsible for the early establishment of disease.

In our study, untreated hearts showed elevated levels of pan-PKC and NF-κB proteins. Treatment with EMPA + PFD decreased chronic myocardial inflammation in HFpEF, as evidenced by reduced pan-PKC protein levels and NF-κB expression in the hearts. Transcriptional data provide a coherent mechanistic explanation for these protein-level changes across three interconnected pathways. First, EMPA + PFD reduced lipid signals that drive PKC overactivation. In untreated HFpEF, elevated pan-PKC protein reflects post-translational overactivation driven by accumulated DAG (Steinberg, 2012), a lipid second messenger generated by phospholipases PLD1 and PLD2, rather than transcriptional upregulation of PKC genes. EMPA + PFD significantly downregulated both PLD1 and PLD2, alongside DGKA, thereby reducing DAG availability and lowering PKC activity (Peivandi et al., 2005). Second, EMPA + PFD normalized calcium signaling via the CaMKII–calmodulin axis. CAMK2D, which was strongly downregulated at disease onset, was significantly restored by EMPA + PFD, indicating recovery from pathological suppression. Concurrently, CALM3 was upregulated, supporting the restoration of physiological CaMKII regulation. Furthermore, PRKCE was strongly upregulated, re-engaging cardioprotective functions that limit NF-κB-driven injury. Third, EMPA + PFD suppressed the receptor for the RAGE–NF-κB axis by targeting S100 calcium-binding protein B (S100B) (Zhou et al., 2024), the most dramatically upregulated gene at disease onset. Because S100B activates NF-κB via the RAGE receptor independently of PKC, its suppression dismantles a parallel inflammatory route. Collectively, the reduction in myocardial inflammation is attributable to a coordinated program that attenuates DAG-driven PKC activation, restores calcium homeostasis, and silences S100B–RAGE signaling.

This coordinated molecular shutdown of inflammatory, lipid, and calcium signaling pathways underpins the significant structural and functional recovery observed *in vivo*. By inhibiting the DAG–PKC and S100B–RAGE pathways, the combination therapy effectively suppresses NF-κB, thereby silencing inflammatory crosstalk and starving the TGF-β cascade. This reduces the local inflammatory environment, prevents extracellular matrix expansion, and leads to a synergistic reduction in deep myocardial fibrosis observed histologically. At the same time, blocking these harmful signaling networks limits pathological hypertrophic growth, aligning with the observed reversal of cardiomyocyte CSA and overall LV mass. Furthermore, restoring intracellular calcium balance through the CaMKII–calmodulin pathway is essential for proper myofilament relaxation. This molecular correction of calcium regulation, along with increased tissue compliance due to reduced fibrosis, underlies the notable, synergistic improvements in diastolic filling (E/A ratio) and overall systolic function (LVEF and stroke volume) observed in the dual-therapy group.

### Sex differences in HFpEF pathophysiology

Sex differences in HFpEF remain underexplored (US FDA, 2014), yet they may influence disease progression and treatment response. In our study, using an HFD + L-NAME-induced HFpEF rat model, significant sex-specific differences were observed: FS was markedly reduced in male rats compared to female rats following HFD + L-NAME (p = 0.0004), with male rats also showing significant FS improvement post-treatment with EMPA + PFD (p = 0.0043 vs. untreated), an effect absent in female subjects. Similarly, LV mass increased more prominently in male rats. These findings align with those of prior studies in spontaneously hypertensive heart failure rats, where FS decreased significantly only in male subjects, despite comparable LV dimensions at failure (Tamura et al., 1999). This sex disparity may stem from estrogen’s protective effects, which mitigate LV hypertrophy and delay heart failure progression in animal models with greater innate hypertrophic reserve (Beaumont et al., 2017; Beale et al., 2018).

We acknowledge that a limitation of the present study is the small sample size regarding sex dimorphism. Although n = 8 per sex provided robust statistical power to detect main treatment effects and synergistic interactions in the pooled cohort, detecting a statistically significant three-way interaction (EMPA × PFD × sex) requires exponentially larger cohorts. Hence, the present study is not sufficiently powered to conclude whether the magnitude of this compartmentalized synergy differs significantly between male and female subjects. Future investigations, powered specifically for sex-based interaction effects, are warranted to explore potential sexually dimorphic responses to this combinatorial therapy.

### Comparison with existing therapies: EMPA + PFD vs. angiotensin receptor–neprilysin inhibitors

Angiotensin receptor–neprilysin inhibitors (ARNis) such as sacubitril/valsartan have emerged as a promising therapy for HFpEF, with recent trials (PARAGON-HF) demonstrating modest reductions in HF hospitalizations but no clear mortality benefit (Solomon et al., 2019). Notably, ARNi exerts antifibrotic effects primarily through natriuretic peptide-mediated cyclic GMP signaling, which inhibits fibroblast activation and reduces ECM deposition (Bozkurt et al., 2023). However, the fibrosis-modulating effects of ARNis appear to be indirect and less pronounced than those observed with direct antifibrotic agents such as PFD. In contrast, PFD directly inhibits TGF-β1 signaling, a central driver of fibrosis in HFpEF, while EMPA enhances cellular metabolism, sodium handling, and vascular function. This dual-action mechanism may provide a more robust strategy for targeting the structural and metabolic abnormalities that characterize HFpEF. Although head-to-head comparisons between EMPA + PFD and ARNi have not been performed, future trials could evaluate whether PFD enhances the antifibrotic effects of ARNi or whether an EMPA + PFD + ARNi combination may provide additional benefits.

### Study limitations and future directions

Considering that SGLT2 inhibitors are now recommended for HFpEF and that PFD is FDA-approved for idiopathic pulmonary fibrosis (Richeldi et al., 2011), our findings suggest the potential for a therapeutic combination. The robust antifibrotic and antihypertrophic synergy observed in our study provides a strong mechanistic rationale for this dual therapy, particularly for patients with a high fibrotic burden.

However, several limitations must be acknowledged. Primarily, HFpEF is a highly heterogeneous syndrome. Although our two-hit model effectively mimics a metabolic-hypertensive phenotype, it cannot fully capture the complexity of human disease, which is often compounded by advanced age and multiple comorbidities, nor does it represent distinct etiologies such as salt-sensitive hypertension. Validating the generalizability of this synergistic effect across diverse preclinical models is essential.

Furthermore, direct clinical translation is limited by the use of supratherapeutic EMPA doses, absence of plasma exposure measurements, and insufficient toxicity assessment regarding metabolic stress, safety, and tolerability of the combination. These factors hinder meaningful evaluation of the benefit–risk profile. Although our findings support compartmentalized synergy, definitive mechanistic validation will require more comprehensive functional studies and integration of key clinical biomarkers (e.g., NT-proBNP, E/e′ ratio, global longitudinal strain, and extracellular volume quantification by cardiac magnetic resonance imaging) (Heidenreich et al., 2022b). This study was also limited by a modest sample size and the lack of a direct comparison with other HFpEF therapies. Future large-scale, comparative studies with extended follow-up are essential to clarify the relative efficacy of EMPA plus PFD and to assess potential mortality benefits.

## Conclusion

In a two-hit HFpEF model, EMPA and PFD co-treatment synergistically ameliorates cardiac dysfunction and pathological remodeling. We identified a compartmentalized synergy localized to the myocardial architecture, driven by simultaneous targeting of the PKC/NF-κB profibrotic cascade and metabolic stress. This dual therapy eradicates cellular hypertrophy and deep fibrosis, restoring diastolic compliance and systolic efficiency and demonstrating that the benefits of SGLT2 inhibition extend beyond systemic diuresis. This study provides proof of concept for a dual metabolic–anti-fibrotic strategy, warranting exploration in future studies for HFpEF treatment.

## Data Availability

The datasets presented in this study can be found in online repositories. The names of the repository/repositories and accession number(s) can be found below: https://www.ncbi.nlm.nih.gov/geo/, GSE277623.
