# Supplementary material for: Empagliflozin–pirfenidone dual therapy improves cardiac function and structure in a preclinical two-hit HFpEF model
Source: Front Pharmacol. 2026 Jun 3;17:1781866. doi: 10.3389/fphar.2026.1781866 (PMC13272355; doi:10.3389/fphar.2026.1781866)
Supplement: Supplementary file 1 [file Supplementaryfile1.docx]

**Supplementary Information**

**
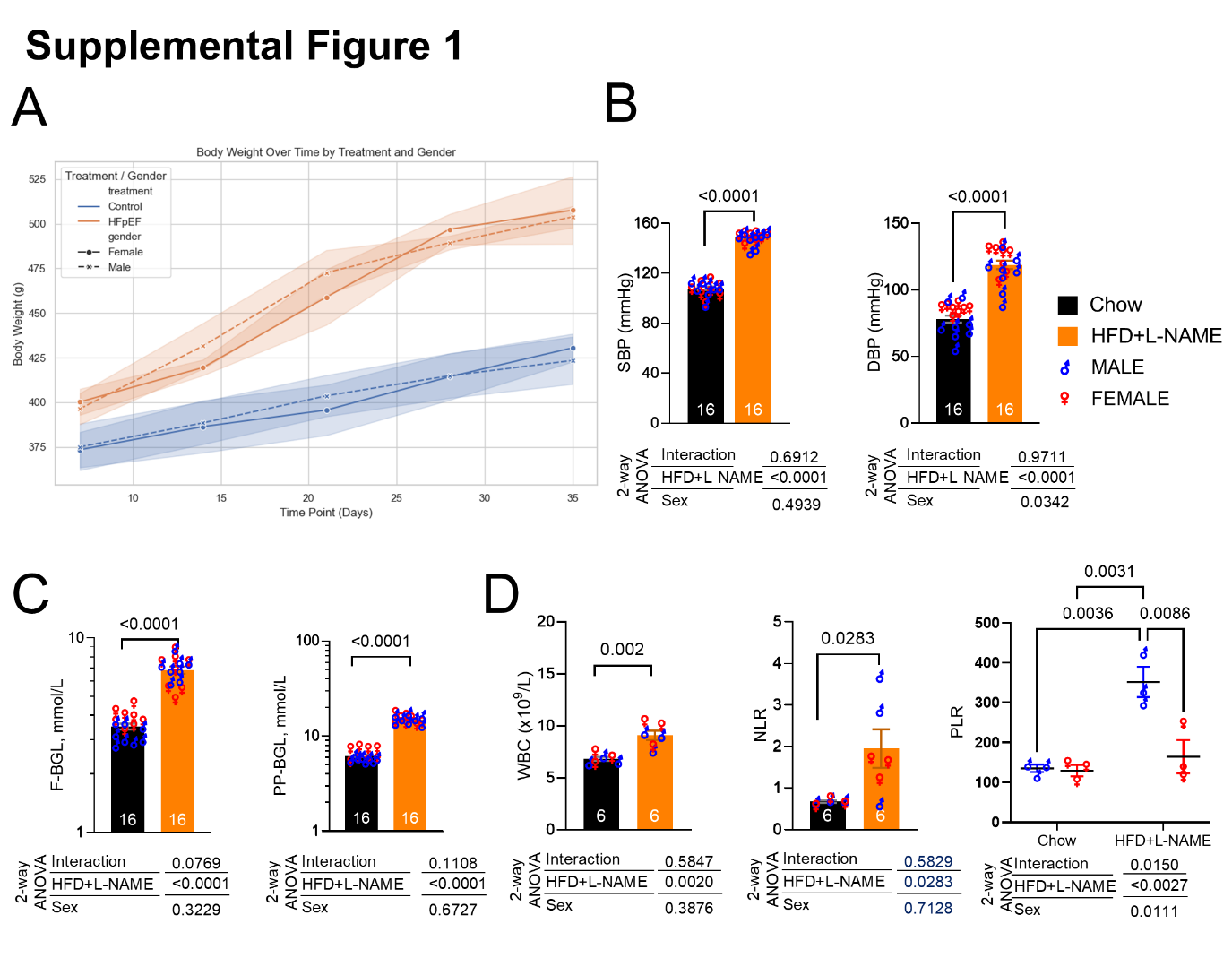
**

**Supplemental Figure 1:** Body weight, blood pressure and hematological parameters in rats-fed with chow and HFD+L-NAME after 5 weeks. (A) Mean body weight (±SEM, shadow) over time (days 7–35) in Control (blue) and HFD+L-NAME (orange) groups by gender (female: solid line with circle; male: dashed line with x; n=8 per subgroup, total n=32 subjects, 160 observations). HFD+L-NAME induces faster weight accumulation versus Control from day 7–35). No gender effects (p≥0.924). Independent two-sample t-test results (parametric, unpaired, two-sided) comparing body weight between Control and HFpEF groups at each time point, with difference between group (HFD+L-NAME vs. control) was presented as unadjusted p-value and deemed significant when p<0.05. (B) Systolic blood pressure (SBP) and diastolic blood pressure (DBP) measurements confirm hypertension in HFpEF rats (p<0.0001 vs. Chow). (C) Fasting (F-BGL) and postprandial blood glucose (PP-BGL), (D) White blood cell (WBC) count, neutrophil-to-lymphocyte (NLR), and (F) platelet-to-lymphocyte ratios (PLR, interaction between sex and HFD+L-NAME, p=0.015). Data are presented as mean ± SEM pooled from both sexes when no interaction between sex and L-NAME was evident, as analyzed using two-way ANOVA with Tukey’s post hoc test, with significance determined at p < 0.05.

**Supplemental Figure 2:**

**
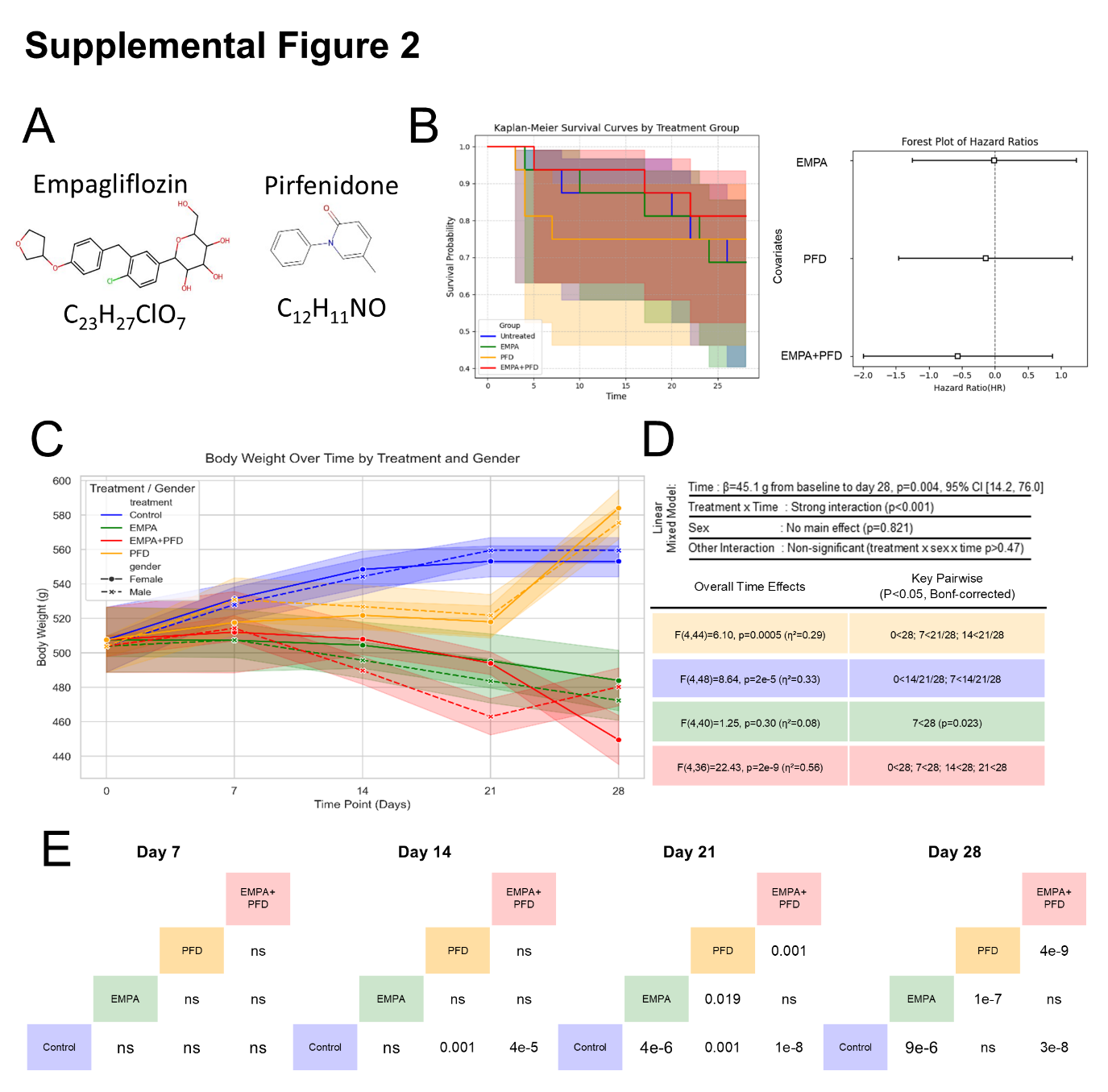
**

1. Chemical structures of Empagliflozin (EMPA) and Pirfenidone (PFD) used in this study. (B) Kaplan-Meier survival analysis shows a trend toward improved survival in EMPA+PFD-treated rats, though statistical significance was not reached (p=0.38, Log-rank test). This analysis was exploratory; the study was underpowered for mortality endpoints, with only 17 events and 73% censoring. Forest plot of hazard ratios (HRs) from a Cox proportional hazards model, adjusted for sex and baseline weight. Circles represent HR estimates for each treatment relative to Untreated; horizontal lines indicate 95% confidence intervals. Although the HR for EMPA+PFD (≈0.53) suggests a possible reduction in mortality risk, the difference was not statistically significant (p=0.38). (C) Mean body weight (± SEM) over time (days 0, 7, 14, 21, 28) for male (solid lines) and female (dashed lines) subjects in each treatment group: Control (black), EMPA (blue), EMPA+PFD (green), and PFD (red). Data from a randomized longitudinal study in rodents (n=64 total subjects; 8 males and 8 females per treatment at baseline, with minor attrition at later time points: 5–8 subjects/group/time). (D) Significant treatment × time interaction from a linear mixed-effects model (p<0.001); (E) Within-treatment time effects (repeated-measures ANOVA, Greenhouse-Geisser corrected where sphericity was violated) suggest significant gains in the Control and PFD groups, while EMPA and EMPA+PFD showed significant net loss over time (adjusted P<0.05). Post hoc pairwise comparisons (Bonferroni-corrected t-tests) indicated significant differences from Control at days 14–28 for EMPA and EMPA+PFD (adjusted p<0.05; bottom). 2e-5 indicates an adjusted p-value of 0.00002.


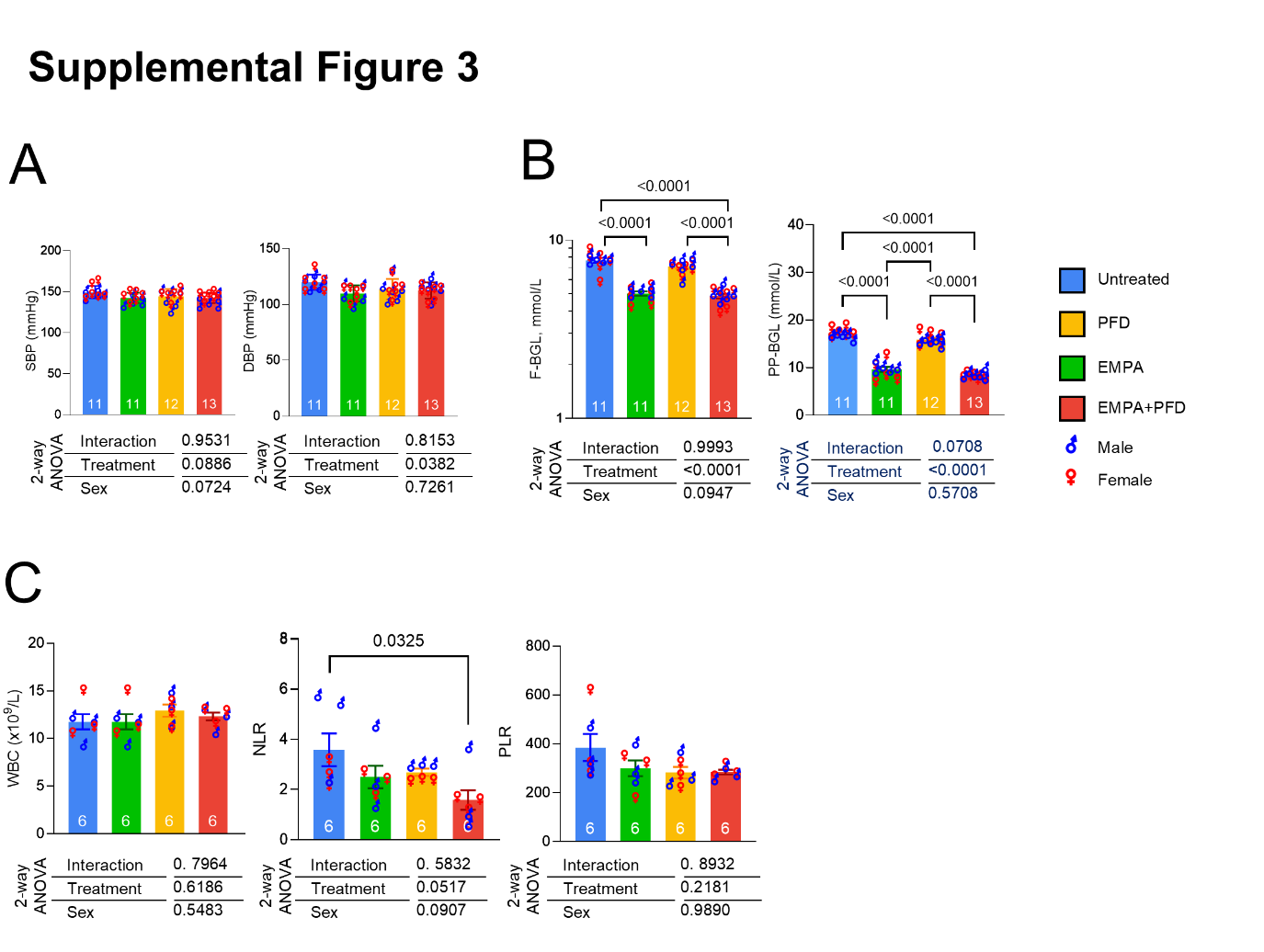


**Supplemental Figure 3:**

Blood pressure and hematological parameters in untreated, EMPA-treated, PFD-treated and EMPA+PFD-treated HFpEF rats after 4 weeks. (A) No difference in SBP and DBP between all groups. (B) EMPA and EMPA+PFD group demonstrated significant reduction in fasting and postprandial blood glucose level (p<0.0001), (C) Hematological WBC and PLR show no difference between treatment, only NLR was marginally lower in EMPA+PFD as compared to untreated control (adjusted p<0.0325) Data are expressed as mean ± SEM, with two-way ANOVA and Tukey’s post hoc test used for analysis.


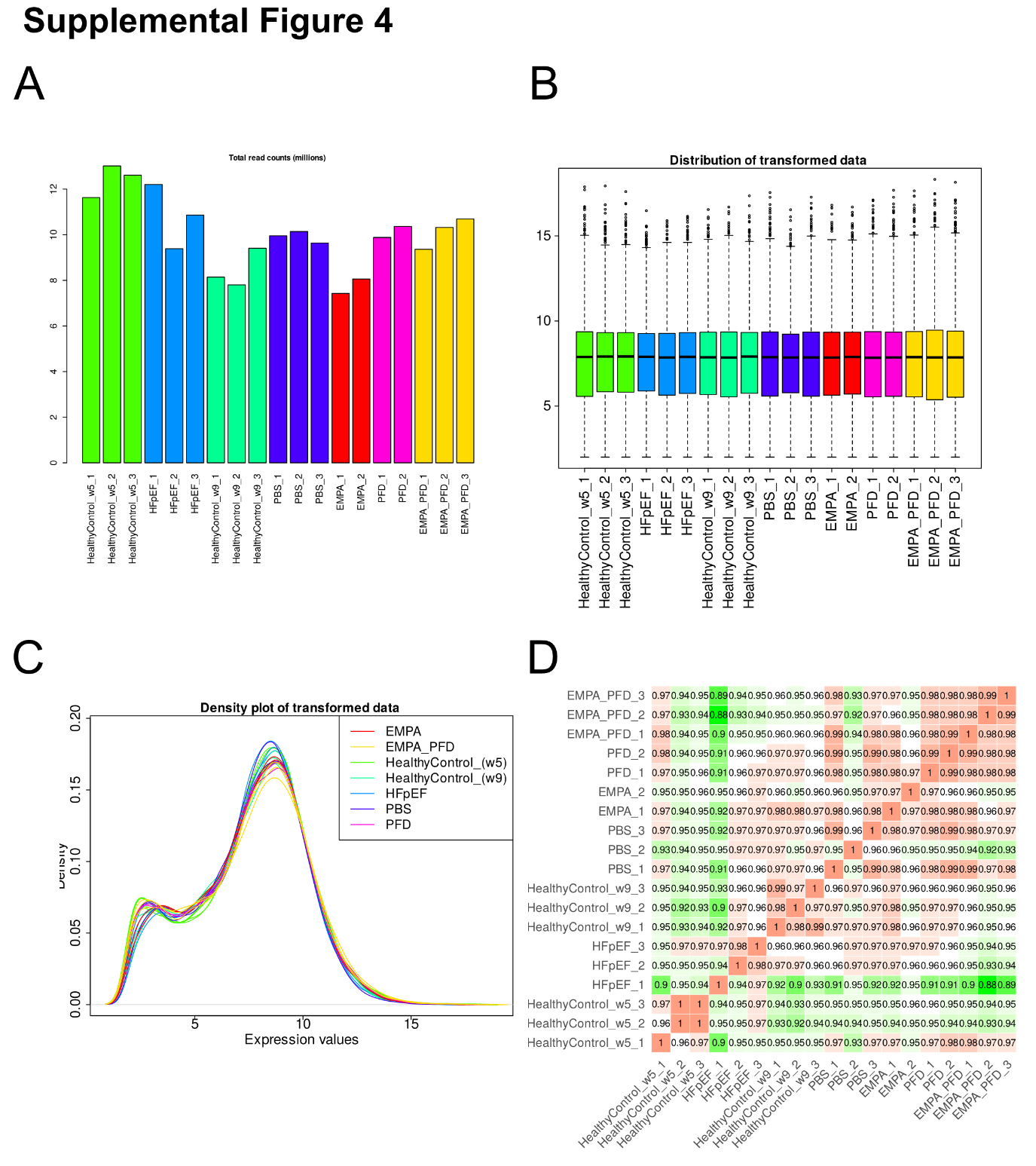


**Supplemental Figure 4:**

RNA sequencing quality control. (A) Total read counts in each library. (B) The distribution and (C) density plots of transformed data. (D) Correlation matrix between two samples.

**
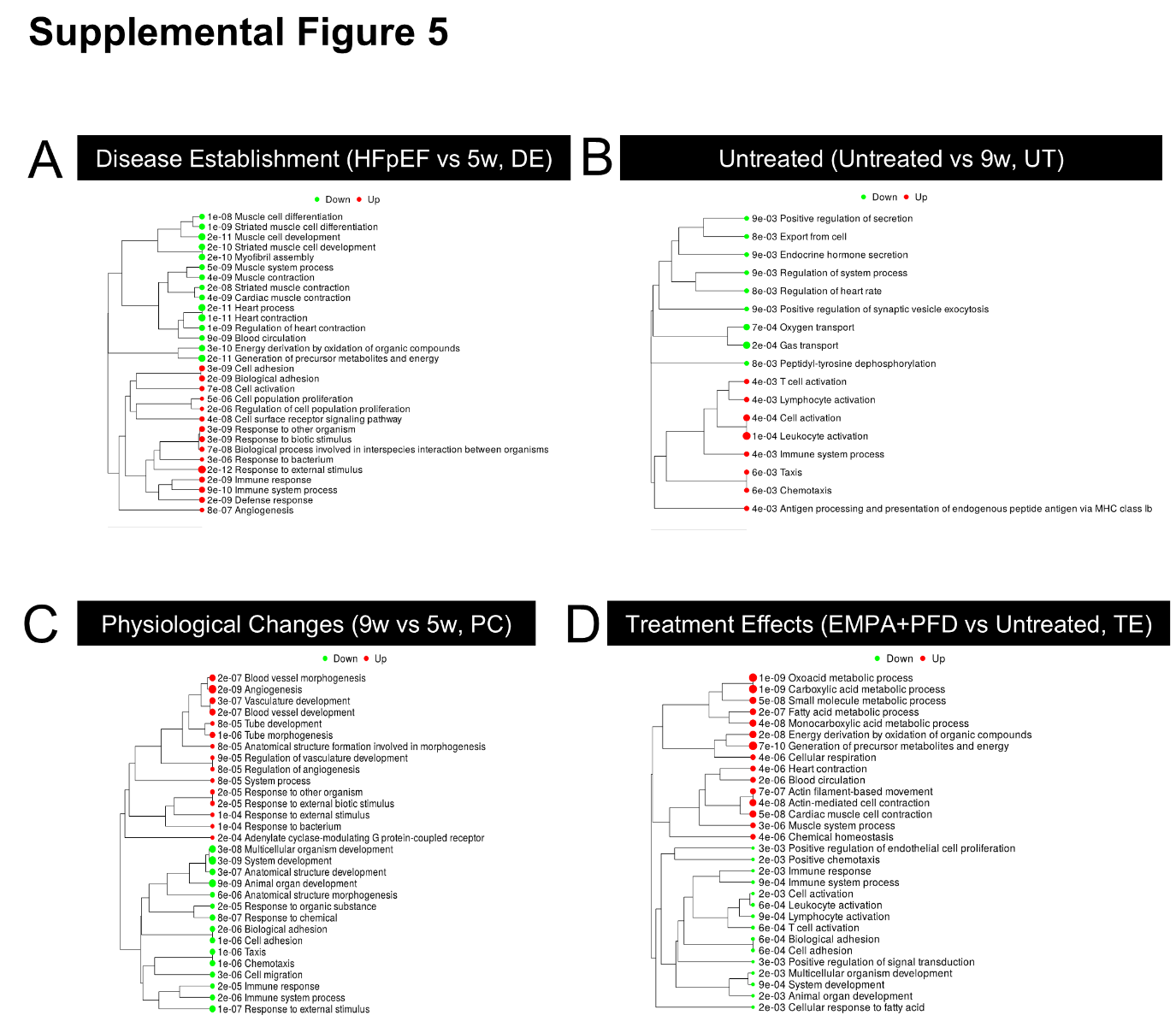
Supplemental Figure 5:**

Transcriptomic analysis in HFpEF rats following PFD, EMPA or PE treatment. (A) Differential expression analysis comparisons (adjusted p value<0.05 and absolute fold change >2). Bar chart presenting the number of upregulated and downregulated DEGs for EMPA+PFD, PFD, EMPA, and Control (9w) vs untreated (left) or vs HFpEF (middle). DEGs comparing EMPA+PFD, PFD, EMPA, untreated, HFpEF, and Control (9w) vs Control (5w) (right). (B) Principal component analysis and (C) Venn diagram analysis of the common DEGs between disease establishment (DE), physiological changes (PC), untreated consequence (UT), and treatment effects (TE). (D) Functional clustering of gene ontology terms for biological processes for both up- and downregulated DEGs for DE, PC, UT, and TE.
